# Kinase inhibitors in organoid media influence *Toxoplasma gondii* growth and development

**DOI:** 10.1128/spectrum.03472-25

**Published:** 2026-04-03

**Authors:** Katie M. Cataldo, Nicole M. Davis, Laura J. Knoll

**Affiliations:** 1 Department of Medical Microbiology & Immunology, University of Wisconsin-Madison 5228, , Madison, Wisconsin, USA; Clemson University, Clemson, South Carolina, USA

**Keywords:** *Toxoplasma gondii*, organoid, media, fibroblast, kinase inhibition

## Abstract

*Toxoplasma gondii* is a widespread parasite that impacts both human and animal health. Increasing use of organoid model systems has made previously challenging aspects of the *T. gondii* lifecycle more accessible. The media for these organoid systems are highly complex with many growth factors and pathway inhibitors that promote stem cell retention or differentiation of the cells. We noticed changes in *T. gondii* growth and development in our intestinal organoid system and wanted to determine if this was driven by cell type or media components. We found that low concentrations of SB202190 (a p38 MAPK inhibitor) and A83-01 (an ALK 4/5/7 receptor inhibitor) are each sufficient to alter *T. gondii* growth even in fibroblast cells. Further investigation with our qPCR panel of *T. gondii* stage markers revealed that these compounds promote bradyzoite cyst development and prime parasites for pre-sexual and sexual stage gene expression. As these complex organoid systems become more common in microbiology research, this study highlights the role of organoid media components in controlling pathogen growth and development.

**IMPORTANCE:**

The use of complex cell culture model systems is becoming more common across many fields. Maintenance of these systems often includes growth factors and inhibitors not found in standard media. These added components have the potential to impact pathogen growth and development and can influence how experimental results may be interpreted. This study improves our understanding of the *Toxoplasma gondii* lifecycle in one of those systems. It also serves as a template for other pathogen researchers to consider influences within their own systems.

## INTRODUCTION

*Toxoplasma gondii* is an obligate intracellular pathogen with a global distribution and a wide host range. The lifecycle of *T. gondii* is complex and includes asexual and sexual stages. Tachyzoites are a fast-replicating, widely disseminated asexual stage that are often cleared by the immune system. Bradyzoites are a slow-replicating, cyst-forming stage that often persists in immune-privileged sites such as the brain and muscle tissue. Asexual stages are present in both the intermediate and definitive hosts. Felines are the only known definitive host and produce a sexual stage called an oocyst. Oocysts are very stable and persistent in the environment, posing a health threat to humans and animals. The strict species-specificity for the feline intestine has made the study of the *T. gondii* sexual stages challenging (1, 2). Because of improvements in organoid culturing technology, our lab previously developed a feline intestinal organoid model to facilitate easier study of sexual stages of *T. gondii in vitro* (3).

The media required for organoid growth and differentiation often include complex cocktails of growth factors and pathway inhibitors (4). Some of these components drive organoids to either remain in a stem cell-like state (5) or to differentiate (6), and others aid in long-term survival of organoid cultures (7). If these varying conditions for organoids are used to grow microbes, it can be difficult to discern if the media component is affecting the microbe directly or if it affects the microbe indirectly by changing the stage of host cell development. Even though components frequently used in organoid media have been found to inhibit viral (8–10) and parasitic growth (11–13), it is uncommon to find published studies that evaluate the impact of media components when adding a pathogen into an organoid model system (14). We noted that organoid media components in feline intestinal organoids had effects on the growth and development of *T. gondii*. To discriminate between the components affecting the differentiation state of the feline cells and the components directly affecting the growth and development of *T. gondii*, we examined the effects of growth factors and inhibitors on *T. gondii* in a simple fibroblast host cell model. We found that organoid media components, especially the kinase inhibitors, affected the growth and development stage gene expression of *T. gondii* grown in fibroblast cells. This study will contribute to building better cell culture models of *T. gondii* development.

## RESULTS

### The organoid media components A83-01 and SB202190 alter parasite morphology

**Fig 1:**
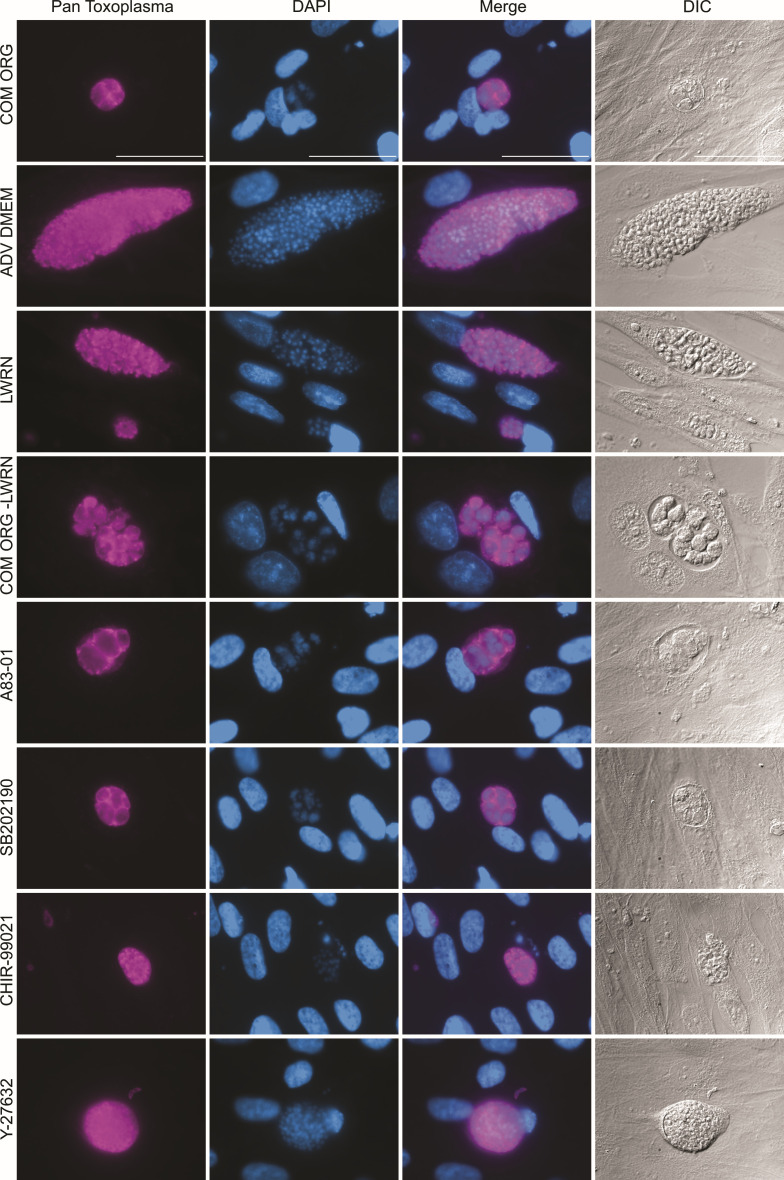
Organoid media components A83-01 and SB202190 alter parasite morphology. HFFs were infected with the *T. gondii* strain ME49 ∆hpt luciferase at a multiplicity of infection (MOI) of 0.1. At 3 hours post-infection (hpi), the media was removed and replaced with the following: complete organoid media (COM ORG), advanced (ADV) DMEM alone, conditioned media components (LWRN), and complete organoid media minus the LWRN conditioned components (COM ORG -LWRN). At 4 days post-infection (dpi), cells were fixed with 100% cold methanol and stained with Pan-*Toxoplasma* antibody (purple), mounted in DAPI (blue), and imaged with differential interference contrast (DIC) microscopy. A representative set of images taken at the same magnification is shown, and the white size bar is 50 µm.

To focus on the role of organoid media components on parasite morphology, we used human foreskin fibroblast (HFF) cells that are well-established to support *T. gondii* growth. We confirmed that the HFFs themselves were tolerant of all treatment conditions (Fig. S1 and Table 1 details media components). We then infected HFFs with *T. gondii* and monitored parasite morphology in each treatment condition (Fig. 1). Parasites grown in Advanced (ADV) Dulbecco’s modified Eagle medium (DMEM)/F12 have distinct outlines showing individual parasites within a large vacuole when stained with a Pan-*Toxoplasma* antibody. In contrast, parasites grown in complete organoid media have irregular structures within their vacuoles. Nuclei as visualized with 4′,6-diamidino-2-phenylindole (DAPI) are also different. In ADV DMEM/F12, they are small, regular, and contained within the individual parasites. In complete organoid media, the nuclei are amorphous and large, although they are still contained within the irregular structures. Parasites grown with the L-WRN conditioned media displayed a normal phenotype, while parasites grown in complete organoid media minus the L-WRN conditioned media displayed irregular morphology. This result was an indicator that the driver of this phenotype was an added component(s) and not an undefined product from the L-WRN cells used to produce the L-WRN conditioned media. The top candidates for these drivers were A83-01 (an ALK/4/5/7 receptor inhibitor, most noted for being upstream of TGFβ), CHIR-99021 (a Wnt activator), SB202190 (a p38MAPK inhibitor), and Y-27632 (an apoptosis inhibitor). When all four components were removed, parasites had a normal morphology. When looking at individual components, CHIR 99021 and Y-27632 did not alter parasite morphology, but treatment with A83-01 and SB202190 induced similar morphological changes to complete organoid media (Fig. 1).

**TABLE 1:** Media list with additives and final concentration found in the material and methods^*a*^

	ADV DMEM/F12	HFF media	LWRN media	Complete organoid media	Reduced organoid media	LWRN conditioned media	Complete organoid -LWRN	A83-01	SB202190	CHIR-99021	Y-27632
ADV DMEM/F12	x		x	x	x	x	x	x	x	x	x
DMEM		x									
Glutamax/glutamine		x	x	x	x	x	x				
Pen/Strep		x	x	x	x	x	x				
Fetal bovine serum (FBS)		x	x	x	x	x	x				
HEPES		x		x	x		x				
B27				x	x		x				
N2				x	x		x				
N-acetylcysteine				x	x		x				
Nicotinamide				x	x		x				
Insulin/selenium/transferrin				x	x		x				
EGF				x	x		x				
A83-01				x			x	x			
SB202190				x			x		x		
CHIR-99021				x			x			x	
Y-27632				x			x				x
Wnt/Rspo/Noggin (L-cell produced)				x	x	x					

^*a*^ “x” indicates that the media contains the reagent listed in the row.

### Parasites grown in complete organoid media, A83-01 and SB202190, display a growth defect

**Fig 2:**
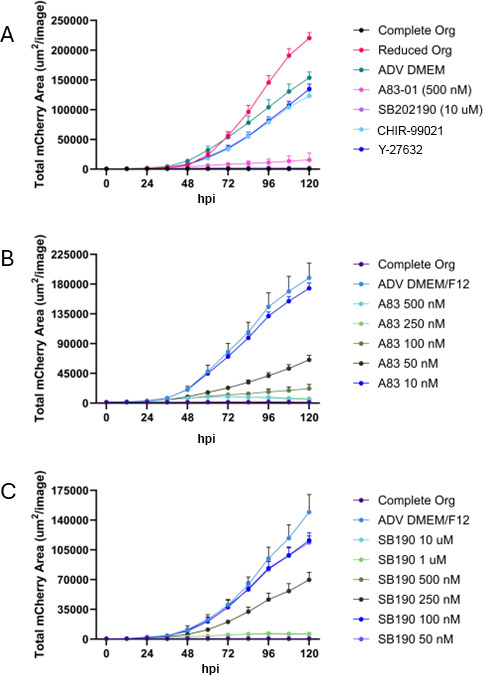
Parasites grown in A83-01 and SB202190 display a growth defect. (**A**) Triplicate wells of HFFs were infected with the *T. gondii* strain PRU Cre mCherry at an MOI of 0.1. After 3 hpi, the media was removed and replaced with the conditions as shown in the legend, and the plate was moved to an IncuCyte Live-Cell Imaging system where red fluorescence and brightfield images were captured every 12 h for 5 days. In advanced DMEM media, the infections were kept that same as panel **A** except concentrations of A83-01 (**B**) and SB202190 (**C**) were varied.

We wanted to examine whether these morphological changes of *T. gondii* had an associated growth defect. Using the IncuCyte Live-Cell Imaging system, we were able to quantify the growth of a fluorescent strain of *T. gondii* (15). *T. gondii* grown in complete organoid media, ADV DMEM/F12 with A83-01, and ADV DMEM/F12 with SB202190 displayed a growth defect relative to ADV DMEM/F12 (representative growth curve Fig. 2A with additional replicates in Fig. S2A). We did not observe a growth defect with the compounds CHIR-99021 and Y-27632 (Fig. 2A; Fig. S2A) and so did not pursue these compounds further. To optimize our organoid model for *T. gondii* growth, we wanted to determine a threshold at which A83-01 (Fig. 2B) and SB202190 (Fig. 2C) still inhibited *T. gondii* growth by performing serial dilutions of the drugs. Inhibition was still observed at 50 nM A83-01 (additional replicates in Fig. S2B), which is 10 times less than the concentration found in complete organoid media. Inhibition was still observed at 250 nM SB202190 (additional replicates in Fig. S2C), which is 40 times less than the concentration found in complete organoid media.

### Parasites retain typical morphological features at low doses of the drugs A83-01 and SB202190

**Fig 3:**
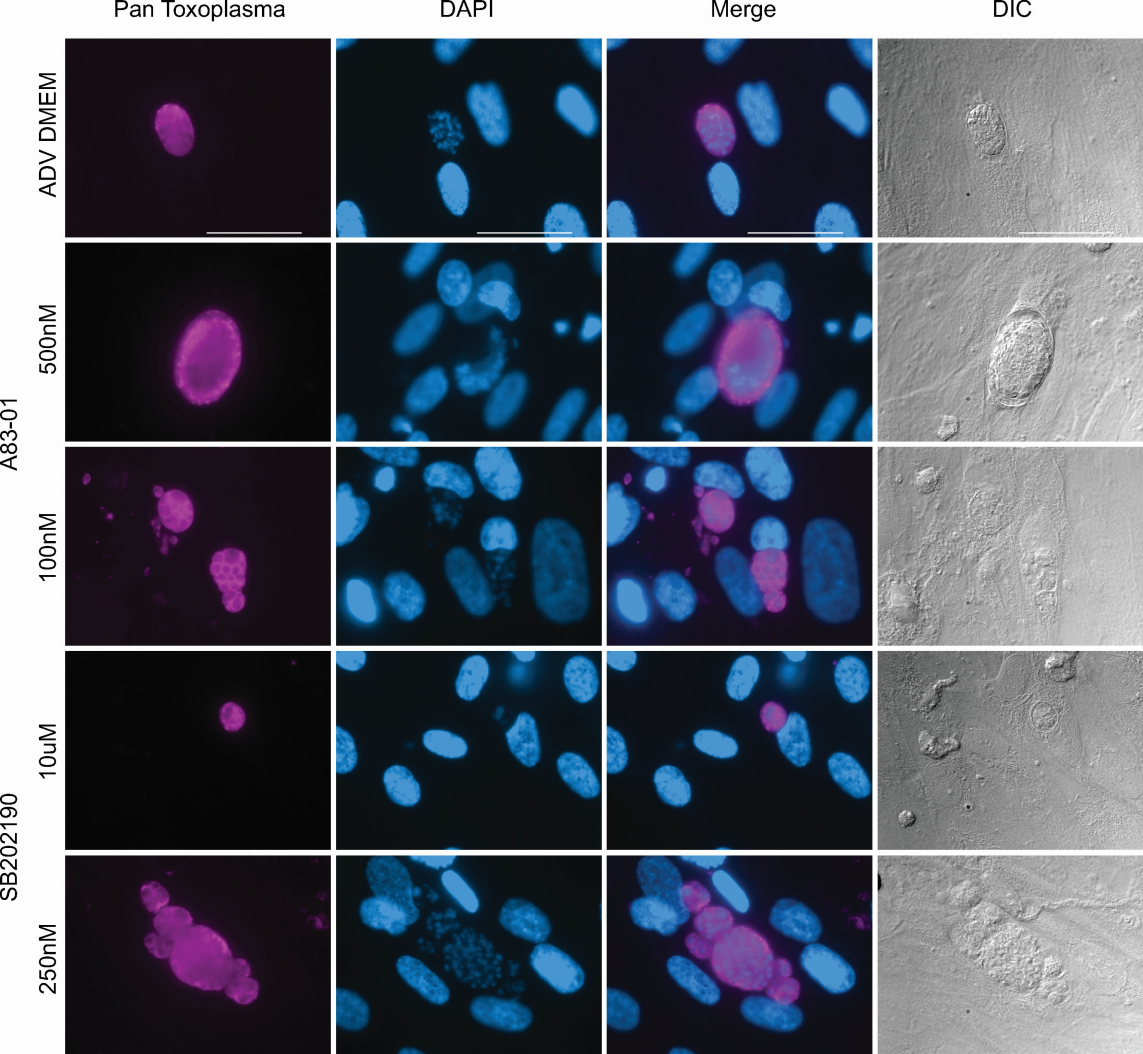
*T. gondii* maintains typical morphology at lower doses of A83-01 and SB202190. HFFs were infected with the *T. gondii* strain ME49 ∆hpt luciferase at an MOI of 0.1. At 3 hpi, the media was removed and replaced with the following: advanced (ADV) DMEM alone or advanced DMEM plus two different concentrations of A83-01 and SB202190. At 4 dpi, cells were fixed with 100% cold methanol and stained with Pan-*Toxoplasma* antibody (purple), mounted in DAPI (blue), and imaged with DIC microscopy. A representative set of images taken at the same magnification is shown, and the white size bar is 50 µm.

Observing the growth inhibition at low doses of the two drugs (50 nM A83-01 and 250 nM SB202190: Fig. 3), we examined if *T. gondii* would still have the irregular morphology found in the high concentrations of the drugs (500 nM A83-01 and 10 µM SB202190). Parasites grown at low doses had typical nuclei, as visualized by DAPI staining. The structures observed in the Pan-*T. gondii* antibody stain more closely resembled the ADV DMEM/F12 condition than the high concentrations of drugs. Upon closer review of the DIC images, there appeared to be potential cyst wall structures forming in some of the conditions, reminiscent of the bradyzoite stage. We performed an immunofluorescence assay (IFA) with Dolichos Biflorus Agglutinin (DBA) to determine if cyst walls were present (Fig. S3). Cyst wall structures were observed in complete organoid media, reduced organoid media, 500 nM A83-01, and 10 µM SB202190 conditions. Wall structures were not observed in ADV DMEM/F12, 50 nM A83-01, and 250 nM SB202190, further indicating that morphologically, the low doses of drugs more closely resemble the ADV DMEM/F12 condition. We quantified the presence of these phenotypes (Fig. S4) by counting 50 vacuoles per condition and identifying them as normal, +DBA, +abnormal nuclei, or both (+DBA and + abnormal nuclei). The ADV DMEM condition had 100% normal vacuoles. The complete organoid, 500 nM A83-01, and 10 μM SB202190 conditions were 90% or greater positive for both DBA and abnormal nuclei. The reduced organoid, 50 nM A83-01, and 250 nM SB202190 were phenotypically normal at least 60% of the time.

We also investigated the timing of drug addition. In the previous figures, drug treatments were added at 3 hpi, which is enough time for parasites to invade host cells but not complete a replication cycle. We added high doses of A83-01 and SB202190 at 24 hpi (Fig. S5), which is enough time for parasites to invade and complete at least one replication cycle. The observations made with a 24 hpi drug treatment are consistent with those made with the 3 hpi drug treatment, showing that the drugs are still affecting the parasite when added after the parasites have begun replicating.

### A83-01 and SB20190 promote bradyzoite gene expression and prime parasites for transition to other stages

**Fig 4:**
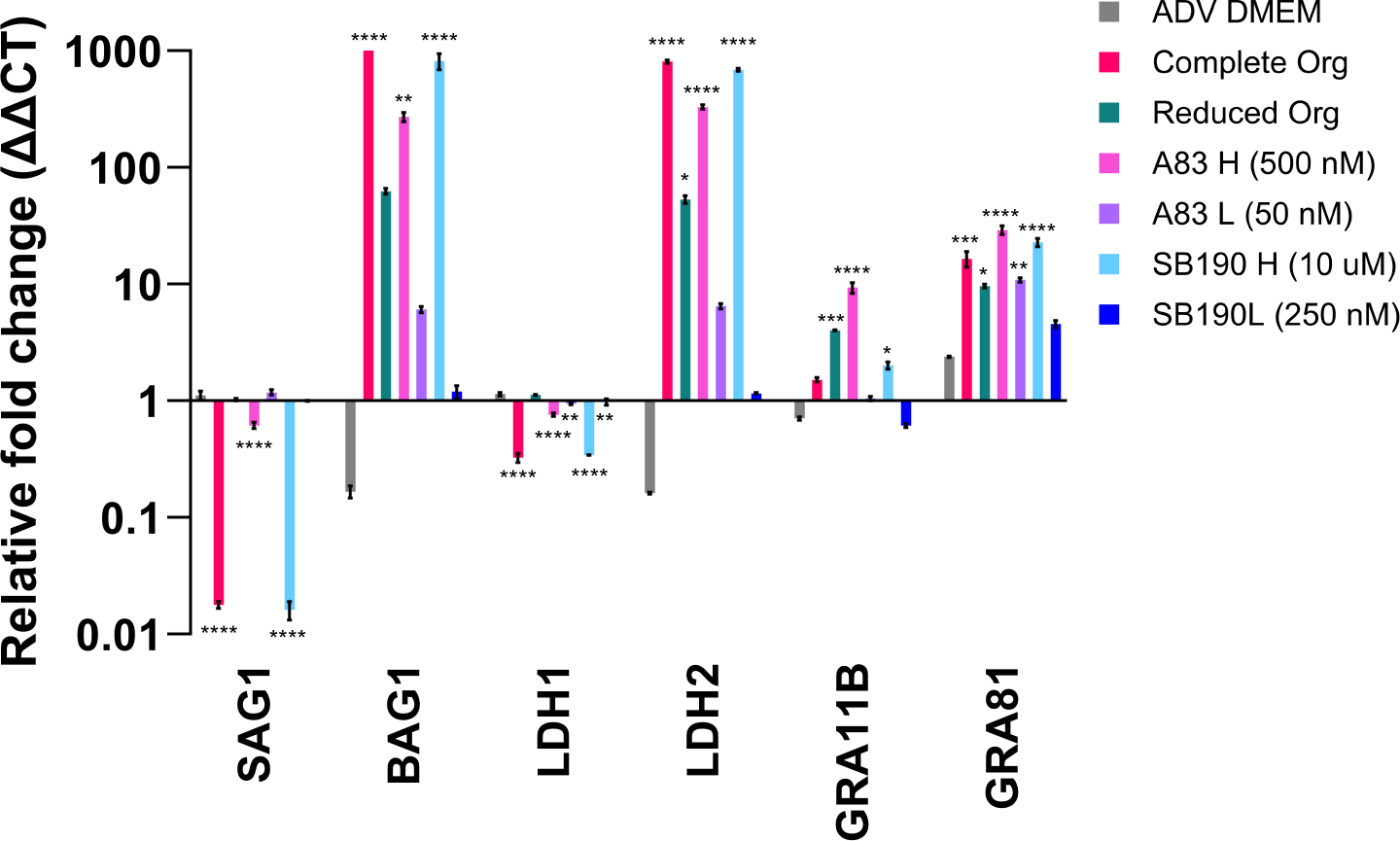
A83-01 and SB20190 promote bradyzoite and pre-sexual gene expression. HFFs were infected with the *T. gondii* strain ME49 ∆hpt luciferase at an MOI of 0.1. At 3 hpi, the media was removed and replaced with the following: DMEM with 10% FBS, advanced (ADV) DMEM, complete organoid (Org), reduced organoid (Org), or advanced DMEM plus two different concentrations of A83-01 (A83) and SB202190 (SB190). At 4 dpi, monolayers were scraped, pelleted, and frozen at −80°C. Cell pellets were resuspended in TRIzol, RNA was isolated, cDNA was produced, and qPCR was performed using primers listed in Table 2. The internal sample control is *T. gondii* TUB1A, and the external sample control is parasites grown in DMEM with 10% FBS. A one-way ANOVA was performed on the technical replicates with * *P* < 0.05, **P < 0.01, ***P < 0.001, and ****P < 0.0001. A representative biological replicate from an *n* = 4 is shown.

To determine if A83-01 and SB20190 were affecting the developmental state of *T. gondii*, we established a qPCR panel of tachyzoite, bradyzoite, and pre-sexual stage markers (Table 2). All comparisons are relative to 10% FBS DMEM, which promotes tachyzoite growth with little bradyzoite and pre-sexual gene expression (representative biological replicate Fig. 4; additional replicates Fig. S6). Most conditions maintain a relatively stable tachyzoite gene expression using surface antigen 1 (SAG1) and lactate dehydrogenase 1 (LDH1) as markers for tachyzoites (Fig. 4; Fig. S6), though statistically significant downregulation is observed in three out of four replicates for the complete organoid and SB202190 High conditions. Using bradyzoite antigen 1 (BAG1) and lactate dehydrogenase 2 (LDH2) as markers for bradyzoites, we saw that bradyzoite gene expression is highest in complete organoid, A83-01 High, and SB202190 High (often reaching statistical significance), but still present in reduced organoid, A83-01 Low, and SB202190 Low (Fig. 4; Fig. S6). A relative increase in pre-sexual markers, GRA11B and GRA81 (Fig. 4; Fig. S6), was seen under all treatment conditions, except ADV DMEM/F12, and statistical significance was often reached in the complete organoid, A83-01 High, and SB202190 High conditions. We examined these parasites for GRA11B protein expression (16) using an IFA, but it was not detected under these conditions (Fig. S7).

**TABLE 2:** Genes, the *T. gondii* stage they are associated with, and the primer sequence used for the qPCR panel

Gene	Sequence 5′−3′	Expression
TUB1A Fwd	CTGCCAATAACTATGCTCGTG	All (housekeeping)
TUB 1A Rev	GGTGAGGATGGAATTGTAGGG	All (housekeeping)
SAG1 Fwd	TGCCCAGCGGGTACTACAAG	Tachyzoite
SAG1 Rev	TGCCGTGTCGAGACTAGCAG	Tachyzoite
BAG1 Fwd	GATGACGTAACCATAGAAGTCGACAAC	Bradyzoite
BAG1 Rev	GCAAAATAACCGGACACTCGCTCAGTC	Bradyzoite
LDH1 Fwd	ATG GCA CCC GCA CTT GTG CAG	Tachyzoite
LDH1 Fwd	GTA AGA GTA CTC AGC ACG G	Tachyzoite
LDH2 Rev	GGAACCATGGGCTACCTTTGTG	Bradyzoite
LDH2 Rev	CTCATACTGGTTTGCGCTCGTCAC	Bradyzoite
GRA11B Fwd	ATCAAGTCGCACGAGACGCC	Merozoite
GRA11B Rev	AGCGAATTGCGTTCCCTGCT	Merozoite
GRA81 Fwd	ATCGTCTTCGGTTCCCTCAT	Merozoite
GRA81 Rev	CTGGAGAGTCTGTTCGATGTCT	Merozoite

### A83-01 and SB202190 act antagonistically toward each other

**Fig 5:**
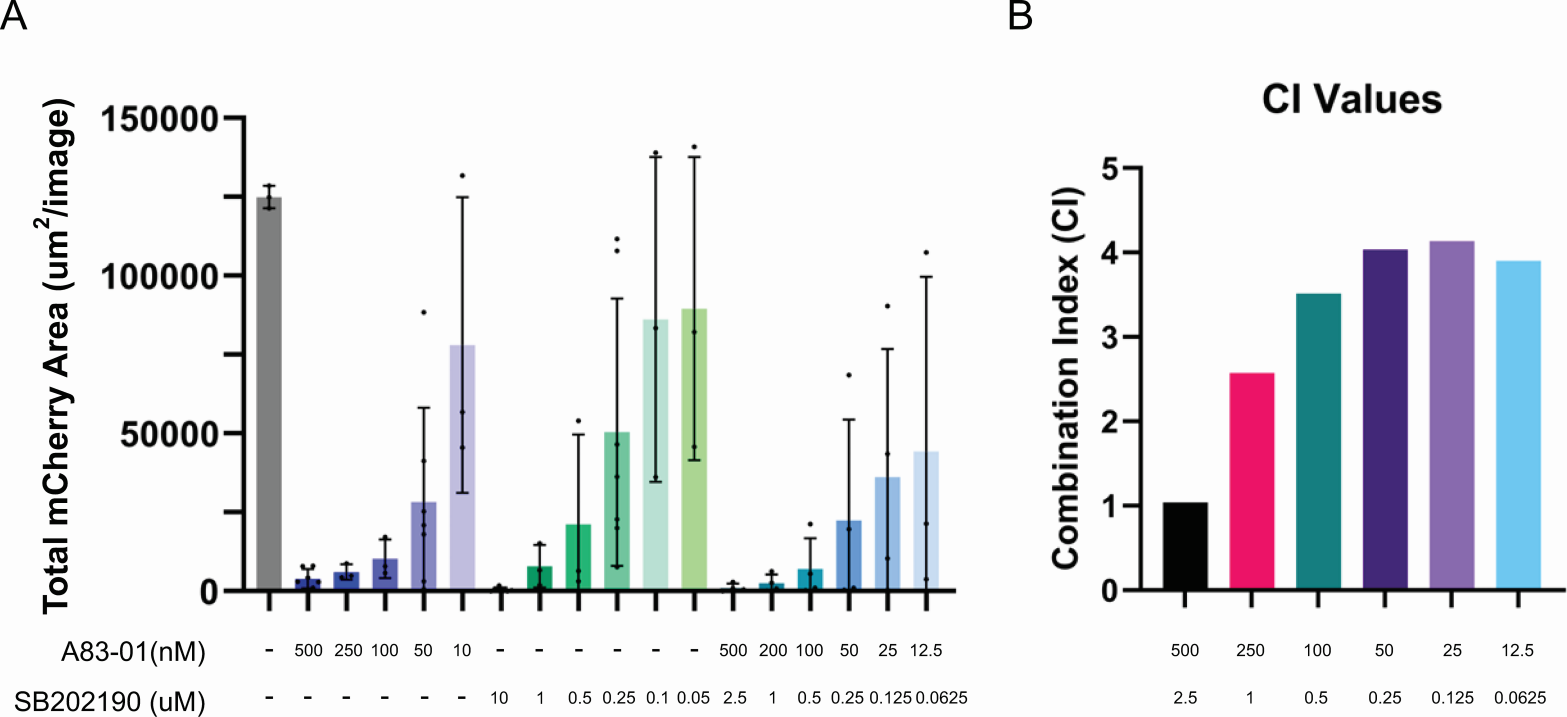
SB202190 and A83-01 act in an antagonistic manner, especially at low doses. (**A**) HFFs were infected with *T. gondii* strain PRU Cre mCherry at an MOI of 0.1. After 3 hpi, the media was removed and replaced with advanced DMEM plus concentrations of A83-01 and SB202190 shown on the y-axis. The plate was subsequently moved to an IncuCyte Live-Cell Imaging system where red fluorescence and brightfield images were captured every 12 h for 5 days. The chart depicts the 4 dpi time points with *n* = 3 and plate controls *n* = 6. (**B**) The CI values were determined using the data from 5A and the Compusyn Computational Tool, where CI > 1 is antagonistic, C = 1 is additive, and CI < 1 is synergistic.

A83-01 and SB202190 are both present in complete organoid media. We wanted to know if these drugs were interacting synergistically (combined effect is greater than total individual effects), additively (combined effect is the same as total individual effects), or antagonistically (combined effect is less than total individual effects). These interactions are determined by a series of mathematical equations for which we used the software tool Compusyn (17, 18). The ratio in which the drugs are present relative to each other is an additional consideration. If this is not kept constant, it represents an additional variable. We chose to use a constant ratio of 1:5 A83-01:SB202190 based on the concentrations (50 nM A83-01 and 250 nM SB202190) identified in Fig. 2 and 3 as impacting parasite growth but not influencing parasite morphology. We used the growth curve value at 4 dpi (Fig. 5A) as our input value for the software. The output of the Compusyn Software is the Combination Index (CI) value. A CI < 1 is synergistic, a CI = 1 is additive, and a CI > 1 is antagonistic. The CI values (Fig. 5B) suggest that A83-01 and SB202190 interact in an antagonistic manner, especially at lower doses.

### Bradyzoites experience similar morphological changes as tachyzoites when treated with A83-01 and SB202190

**Fig 6:**
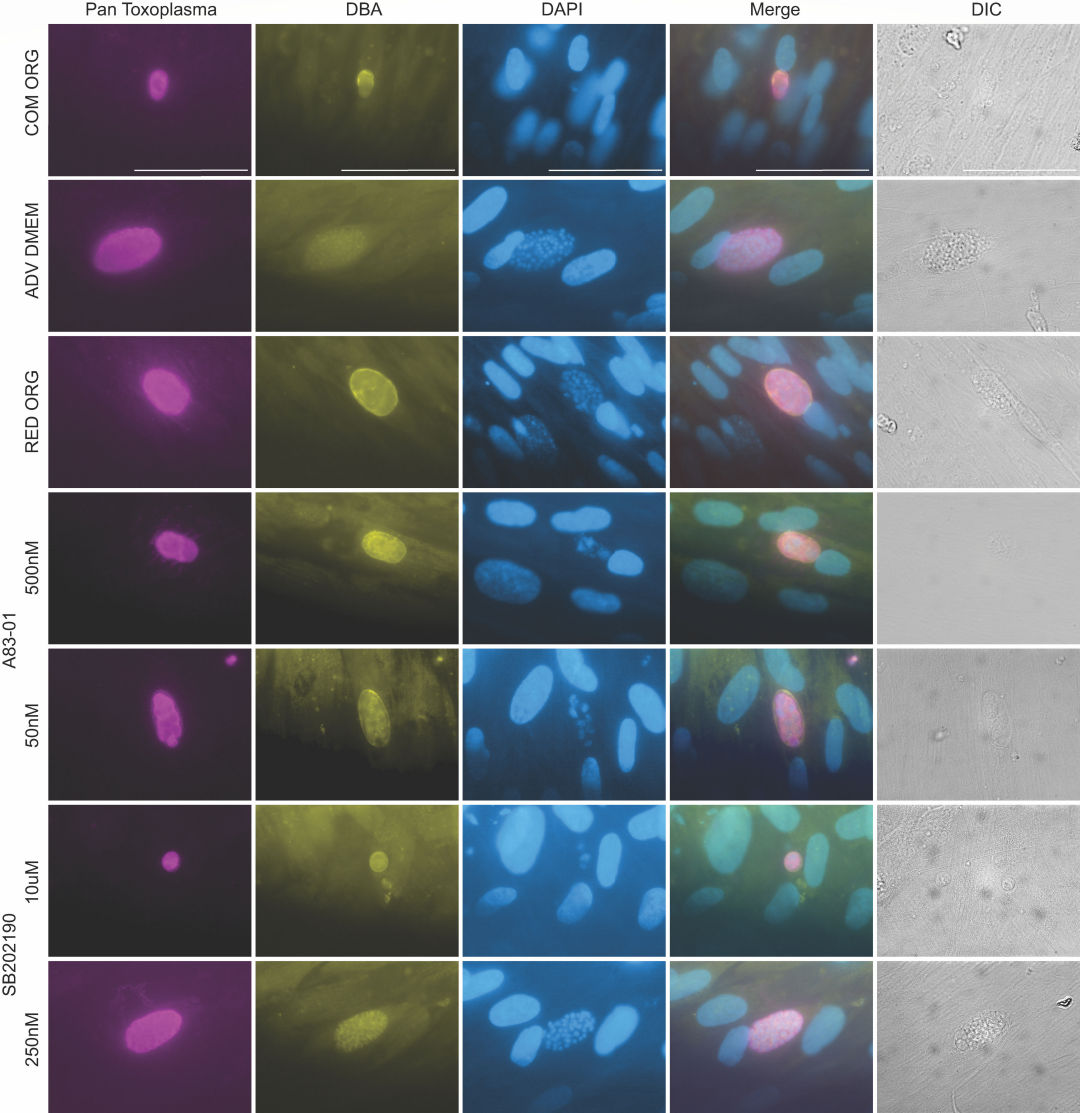
Bradyzoites experience similar morphological changes as tachyzoites under SB202190 and A83-01. Confluent HFFs were infected with *in vitro* bradyzoites from the *T. gondii* strain ME49 ∆hpt luciferase at an MOI of 0.5. At 3 hpi, the media was removed and replaced with the following: complete organoid (COM ORG), advanced DMEM, reduced organoid (RED ORG), or advanced DMEM plus two different concentrations of A83-01 and SB202190. At 5 dpi, cells were fixed with 100% cold methanol and stained with Pan-*Toxoplasma* antibody (purple), DBA (yellow), mounted in DAPI (blue), and imaged with DIC microscopy. A representative set of images taken at the same magnification is shown, and the white size bar is 50 µm.

Bradyzoite stage switching dynamics are important for the sexual cycle. We wanted to determine if beginning these assays with bradyzoites would influence the parasites’ response to these drugs. *In vitro* bradyzoites were generated using high pH media and 33°C (19). These bradyzoites were allowed to invade HFFs for 3 h before the media was changed to test the various components. After 5 days, we performed an IFA using Pan-*T. gondii* antibodies, DBA, and DAPI (Fig. 6). Cyst wall structures were observed in all conditions except ADV DMEM/F12 and SB202190 Low. The irregular vacuole structures were observed in complete organoid media, both concentrations of A83-01 and SB202190 Low, and abnormal nuclei were also observed in those conditions. We observed variability in the overall abundance of vacuoles across conditions when quantifying normal, +DBA, +abnormal nuclei, and both (+DBA and abnormal nuclei). Specifically, all treatments except ADV DMEM/F12 had fewer than 40 total vacuoles across all available coverslips (also see Fig. S8 legend). As such, only raw counts (Fig. S8) were included for analysis. Overall, starting the assay with bradyzoites resulted in similar observations to starting with tachyzoites.

### Media conditions determine whether bradyzoites remain as bradyzoites or switch to tachyzoites

**Fig 7:**
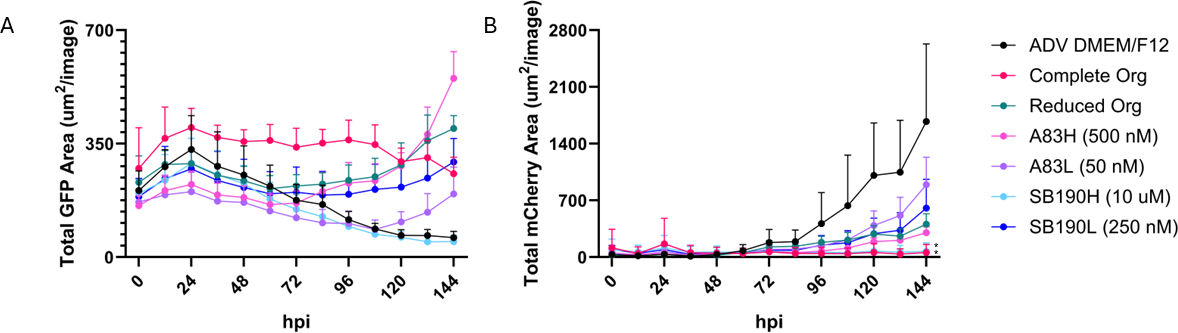
Growth curves reflect a maintenance of bradyzoite characteristics under drug treatment. HFFs were grown to confluency and infected with *in vitro* bradyzoites from the *T. gondii* strain (EGS). After 3 hpi, the media was removed and replaced with the following: advanced DMEM, complete organoid (Org), reduced organoid (Org), or advanced DMEM plus two different concentrations of A83-01 (A83) and SB202190 (SB190). The plate was subsequently moved to an IncuCyte Live-Cell Imaging system where red fluorescence, green fluorescence, and brightfield images were captured every 12 h for 6 days. (**A**) Representative plot of the GFP fluorescence from an *n* = 3. (**B**) Representative plot of mCherry fluorescence from the same replicate as A, *n* = 3. Statistical analysis was performed using a one-way ANOVA followed by post hoc Dunnett’s test comparing the area under the curve of ADV DMEM media to all other media conditions. * Indicates *P* < 0.05.

By IFA alone, we were unable to determine if the bradyzoites had remained bradyzoites over the duration of that 5-day infection or if switching had occurred. We were able to obtain a dual fluorescent parasite strain (EGS Type I/III with LDH2-GFP SAG1-mCherry), which has GFP under a bradyzoite promoter, LDH2, and mCherry under a tachyzoite promoter, SAG1 (20). This dual reporting system allows us to produce growth curves from the IncuCyte using both GFP (bradyzoite) (Fig. 7A) and mCherry (tachyzoite) (Fig. 7B) gene expressions. Fig. 7A and B are representative plots, with replicates available in Fig. S9. A peak in bradyzoite expression of GFP levels is observed around 24 hpi, and complete organoid media maintains relatively steady levels for the duration of the experiment, but ADV DMEM/F12 and SB202190 Low decrease GFP expression for the remainder of the experiment. This decrease in GFP is roughly correlated with an increase in mCherry, indicating switching to tachyzoites and replication. All other conditions experienced an increase in GFP at 72 hpi or later. mCherry levels are not readily detected until 72 hpi. By 144 hpi, complete organoid media remains low, ADV DMEM/F12 is generally high, and both A83-01 and SB202190 have increased, with the lower concentrations having more signals than the higher concentrations. We used area under the curve as a metric to determine which media conditions differed significantly from the ADV DMEM/F12 control condition. By this metric, only parasites in the complete organoid media and high doses of drugs had significantly less mCherry expression than control parasites in ADV DMEM/F12. Taken together, these results indicate that in ADV DMEM/F12, bradyzoites switch to tachyzoites, while in complete organoid media, bradyzoites seem to remain as bradyzoites.

### High doses of A83-01 and SB202190 promote maintenance of bradyzoites and prime for transition to presexual stages

**Fig 8:**
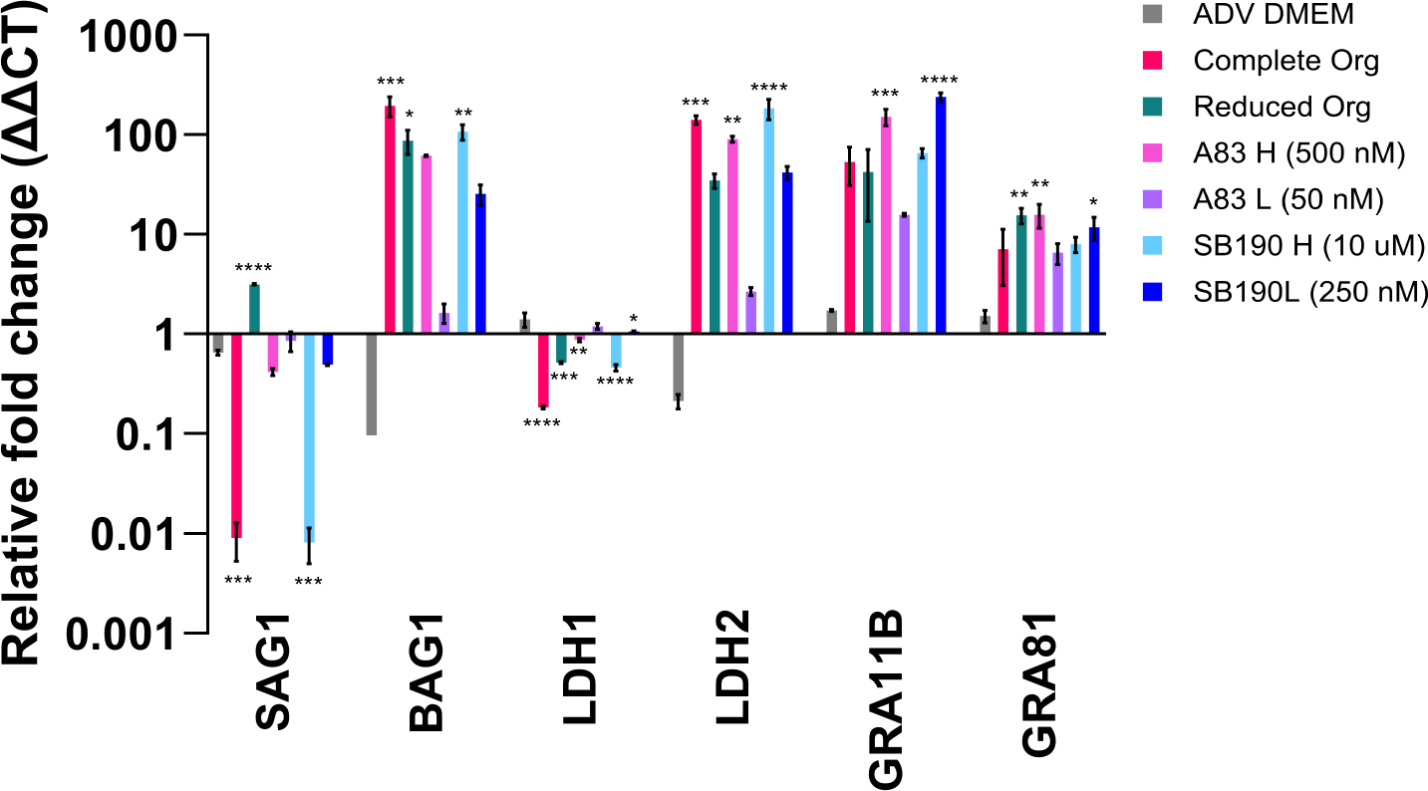
High doses of A83-01 and SB202190 promote the maintenance of the bradyzoite stage and prime for transition to pre-sexual stages. HFFs were grown to confluency and infected with *in vitro* bradyzoites of the *T. gondii* strain ME49 ∆hpt luciferase at an MOI of 0.5. After 3 hpi, the media was removed and replaced with the following: advanced DMEM, complete organoid (Org), reduced organoid (Org), or advanced DMEM plus two different concentrations of A83-01 (A83) and SB202190 (SB190). At 5–6 dpi, monolayers were scraped, pelleted, and frozen at −80°C. Cell pellets were resuspended in TRIzol, RNA was isolated, cDNA was produced, and qPCR was performed using primers listed in Table 2. The internal sample control is *T. gondii* TUB1A, and the external sample control is parasites grown in DMEM with 10% FBS. A one-way ANOVA was performed on the technical replicates with *P < 0.05, **P < 0.01, ***P < 0.001, and ****P < 0.0001. A representative biological replicate from an *n* = 4 is shown.

To further examine bradyzoite stage conversion when grown in the various conditions, we used the established qPCR panel of tachyzoite, bradyzoite, and pre-sexual stage markers (Table 2). All conditions are relative to 10% FBS DMEM, which promotes tachyzoite growth (representative biological replicate Fig. 8; additional replicates Fig. S10). Tachyzoite gene expression was generally significantly downregulated in complete organoid media and high drug doses (Fig. 8; Fig. S10) but was relatively stable in other conditions. Bradyzoite gene expression remained upregulated at 5 dpi in all conditions except ADV DMEM/F12 and low drug doses. An increase in pre-sexual markers was seen under all treatment conditions, except ADV DMEM/F12, and was statistically significant at high doses of the drugs. Using an IFA, we examined these parasites for GRA11B (16) protein expression, but it was not detected (Fig. S11).

## DISCUSSION

We found that two components of complete organoid media A83-01 and SB202190 inhibit *T. gondii* growth and cause unusual morphological changes. In general, these components seem to promote switching to bradyzoites when starting with tachyzoites or maintenance of bradyzoites when starting with bradyzoites. Interestingly, whether the infection was started with tachyzoites or bradyzoites, pre-sexual stage gene expression was observed, indicating that these components also seem to prime parasites for pre-sexual stage gene expression.

A83-01 and SB202190 have antiparasitic potential. The reduced growth and abnormal morphology of both tachyzoites and bradyzoites at high doses of both drugs indicate that they are inhibitory to *T. gondii*. SB202190 is a p38 MAPK inhibitor, and A83-01 is an ALK 4/5/7 receptor inhibitor; both classes of inhibitors have been shown to inhibit TgMAPKL1 (13). As *T. gondii* is an intracellular parasite, the role of these compounds on the host cells in the context of infection is not fully understood but will be an important avenue of investigation in the future.

To examine the interaction of A83-01 and SB202190, we calculated the CI values using Compusyn (17). The CI of A83-01 and SB202190 was close to 1 (additive effect) at high concentrations but greater than 1 (antagonistic effect) at lower concentrations of the drugs (Fig. 5). Reported IC_50_ values for these drugs (21, 22) are closer in range to the lower concentrations in our synergy assay. These results likely indicate that at low concentrations, the drugs are binding to their intended targets, while at high concentrations, the drugs are having off-target effects. Similarly, 10× less of A83-01 and 40× less of SB202190 were still inhibitory toward parasite growth (Fig. 2). The strong phenotype of TgMAPKL1 inhibition is likely masking roles of host cellular kinase pathways in *T. gondii* growth and development.

It is extremely difficult for drugs to penetrate the bradyzoite cyst wall. It is likely that some of the impacts observed on the bradyzoites were a result of host cell kinase inhibition. To further understand the role of host cell kinases in bradyzoite maintenance and control, it would be ideal to have a knockout mutant of TgMAPKL1 as TgMAPKL1 is suspected to be the main driver of the phenotypes in related compounds (11–13). However, TgMAPKL1 has been scored as an essential gene in transcriptomics studies (23), but there has been some limited success in making knockdown mutants of TgMAPKL1 in the RH strain (13, 24). RH is impaired in its ability to develop into bradyzoites in cell culture and is lethal for all mice during acute infection, so it would be useful to develop a TgMAPKL1 knockdown in a type II strain (25) that maintains capability of forming bradyzoites and retains the potential to be used for *in vivo* studies.

Using stressors to switch tachyzoites to bradyzoites is common in the field, though there is growing evidence that they have transcriptional differences from animal tissue-derived bradyzoites (26, 27). Methods used to generate *in vitro* bradyzoites include temperature, pH, and nutrient deprivation. To produce our *in vitro* bradyzoites, we used a combination of pH and temperature (19). Previous studies using a protein kinase G inhibitor (28–30) support the potential to use drugs like A83-01 and SB202190 to create *in vitro* bradyzoites with a few advantages. Single-drug treatments of A83-01 and SB202190 seem to be well tolerated by host cells, which is not always the case with some of the other methods. This research may open up the possibility of studying bradyzoites in more relevant host cells. A83-01 and SB202190 also seem to hold parasites in a bradyzoite state for longer periods of time. While terminal points were taken at 4–6 dpi for IFAs and qPCR based on the survival of untreated cells under infection, it was possible to carry out the IncuCyte experiments to 10 dpi without lysis of the treated monolayers or replacement of the media (data not shown). This extension could open the door to more biologically relevant studies of bradyzoite biology.

The qPCR panel provides a quick and simple assessment of gene expression across various *T. gondii* life stages. Studies of pre-sexual and sexual stages of *T. gondii* have been limited, especially *in vitro* (3, 31, 32). Having the ability to quickly analyze gene expression will help with the understanding of triggers for these pathways. Whether the starting material was tachyzoites (Fig. 4; Fig. S6) or bradyzoites (Fig. 8; Fig. S10), we observed that gene expression was increased in two pre-sexual stage markers, but this increase in gene expression did not increase protein expression of GRA11B (Fig. S7 and S11). The paradigm in the *T. gondii* field is that tachyzoites must first convert to bradyzoites before entering the pre-sexual cycle. Nearly all cats excrete oocysts 3–10 days after ingestion of bradyzoites, whereas <30% of cats excrete oocysts after the ingestion of tachyzoites or oocysts, and >13 or >18 days are required, respectively (33, 34). This decrease and delay in shedding was thought to be caused by the necessity of sporozoites and tachyzoites to develop into bradyzoites before sexual development could occur (35). Under that paradigm, we might have expected that starting with bradyzoite material would result in higher expression of pre-sexual markers because parasites were already primed for pre-sexual gene expression. While GRA11B expression was slightly higher when we started with bradyzoites vs tachyzoites, the fact that it was not substantially higher might mean that the pathways A83-01 and SB202190 are involved in may be equally active in tachyzoites and bradyzoites. Additionally, translational repression has been observed within the Apicomplexan phylum during the maturation processes (36) and stress responses (37), which may also play a role in the absence of GRA11B protein expression. Further study of the reduced organoid media condition may help differentiate some of these possibilities. The reduced organoid media does not contain either A83-01 or SB202190 and still has a relatively high expression of the pre-sexual stage gene markers. This result is an indication that other components of the media may be influencing the priming for pre-sexual stage gene expression and could provide an additional tool for understanding triggers of this understudied pathway.

Based on a greater understanding of the impacts A83-01 and SB202190 have on the parasites, we plan to use this to optimize our intestinal organoid model. Because parasites seem primed to express pre-sexual genes, we will investigate further some potential triggers of the pre-sexual stages. Kinase regulation of both the host and parasite seems to play an important role in *T. gondii* growth and development and will be an interesting avenue for future studies. In this study, we used relatively immature *in vitro* bradyzoites because we were interested in the potential of A83-01 and SB202190 to prevent transitions from bradyzoites back to tachyzoites. Future studies seeking to evaluate the antiparasitic potential of A83-01 and SB202190, especially against mature cysts in the brain or tissues, may choose instead to begin assays with mature bradyzoites isolated from the brain.

As the microbiology community embraces increasingly complex *in vitro* systems such as organoids and organ on a chip, this study should serve as a cautionary tale. Media components can have direct impacts on pathogenic organisms. Not accounting for potential inhibitors or activators in the media could result in misunderstanding of the importance of host cell development stage, infection processes, or inaccurate conclusions about drug effects in the system. Commercial, pre-made medias with proprietary components are available, but this may not be the best course of action for troubleshooting less established systems of infection.

## MATERIALS AND METHODS

### Tachyzoite culture

All cultures were maintained at 37°C, 5% CO_2_, in a humified incubator unless otherwise specified. *T. gondii* tachyzoites were cultured in human foreskin fibroblasts (ATCC SCRC-1041) in Dulbecco’s modified Eagle’s medium (Gibco) supplemented with 10% fetal bovine serum, 2 mM L-glutamine, and 1% penicillin-streptomycin. Parasites were counted and passaged weekly. To reduce the possibility of tissue culture-specific characteristics, low passages of parasites (less than 25) were used when possible. Strains used were as follows: ME49 Δhpt Luciferase, Pru Cre mCherry, and EGS LDH2p-GFP/SAG1p-mCherry.

### Bradyzoite culture

Confluent HFFs were infected with 1 × 10^6^ tachyzoites. After 2 hours, media was removed and replaced with differentiation media: RPMI 1640 without sodium bicarbonate and L-glutamine (R7755, Sigma-Aldrich), 1% FBS, and 40 mM HEPES, with the pH adjusted to 8.1. The flask cap was completely tightened to avoid gas exchange, and infected cells were incubated at 33°C, at ambient air conditions for 10–14 days prior to use.

### Bradyzoite excystation and pepsin activation

Media was removed from bradyzoite culture and replaced with 1% NaCl pH 2.1 solution and pepsin in a 1:1 ratio. The cell monolayer was scraped and resuspended by pipetting to break up large chunks. Digestion was allowed to proceed for 30 min before being neutralized by an equal volume of 1% Na_2_CO_3_ solution. To the neutralized solution, an equal volume of culture media is added (DMEM, 10% FBS, 2 mM L-glutamine, and 1% pen/strep), followed by centrifugation at 250 × g for 10 min. The supernatant was aspirated, resuspended, and then counted.

### Media formulations

Media reagents with final concentrations are as follows: ADV DMEM/F12 (Gibco), 1× Glutamax (Lifetech), 1× HEPES (Lifetech), 1× B27 (Lifetech), 1× N2 Supplement (Lifetech), 1 mM N-acetylcysteine (Sigma-Aldrich), 10 mM nicotinamide (Sigma-Aldrich), 1× insulin/selenium/transferrin (Gibco), 50 ng/mL human EGF (R&D Systems), 2.5 μM CHIR-99021 (Selleckchem), 10 μM Y-27632 (STEMCELL Technologies), 500 nM A83-01 (AGscientific), 10 μM SB202190 (Selleckchem), and 50% conditioned media from the L-WRN cell line (ATCC CRL 3276).

### Immunofluorescence

HFF coverslips were fixed and permeabilized with 100% ice-cold methanol for 10 min and then blocked at room temperature with 3% BSA in PBS. The primary antibody was incubated at room temperature for 2 h with 3% BSA in PBS: *Toxoplasma gondii* polyclonal antibody (Invitrogen Cat# PA1-7252) at a ratio of 1:500. For the GRA11B antibody (Adrian Hehl [16]), coverslips were incubated in the undiluted GRA11B hybridoma supernatant. Secondary and conjugated antibodies were incubated at room temperature for 1 hour with 3% BSA in PBS: Alexa Fluor 594 goat anti-rabbit (Invitrogen Cat# A11012) 1:500, Alexa Fluor 488 goat anti-mouse (Invitrogen Cat# A11001) 1:500, and DBA Fluorescein (Vector Labs Cat# FL-1031) 1:200. Cell nuclei were stained with DAPI (Sigma Cat# D9564-10MG). For GRA11B antibody, (Slides were mounted with Vectashield antifade mounting medium (Vector Labs Cat# H-1000). Samples were imaged on Zeiss Axioplan III equipped with a triple-pass (DAPI/fluorescein isothiocyanate [FITC]/Texas Red/DIC) emission cube, differential interference contrast optics, and a monochromatic Axiocam camera operated by Zen software (Zeiss).

### IncuCyte growth curves

HFFs were plated in 48-well plates (nunc) and allowed to reach confluency. Cells were infected with tachyzoites at an MOI of 0.1 or bradyzoites at an MOI of 0.5. At 3 hpi, media was removed and replaced with experimental conditions. The plate was then moved to the IncuCyte Live- Cell Imaging System (Sartorius) and set to scan every 12 hours for 7 days (tachyzoites) or 10 days (bradyzoites), 16 images per well, at 20× magnification. Images were quantified using the IncuCyte Analysis Software (Sartorius). AUC analysis was performed in GraphpPad Prism version 10.3.1 using default parameters (baseline = 0, ignoring peaks less than 10% of the distance from minimum to maximum Y, and defining peaks as being above, not below, baseline).

### qPCR analysis

Cell monolayers were scraped, pelleted at 500 × g for 5 min, and frozen at −80°C. Cell pellets were resuspended in TriRIzol. Total RNA was isolated using phenol/chloroform extraction and DNase- treated. cDNA was produced using Superscript III (Thermo Fisher) and oligoDTs (Thermo Fisher). Primers (3, 38, 39) from prior literature or designed using the IDT PrimerQuest tool used for the panel are described in Table 2. qPCR was performed with PowerSYBR Green PCR Master Mix (Applied Biosystems) and run on a QuantStudio 7 real-time PCR system (Applied Biosystems). The ΔΔCT method was used to determine relative gene expression, with HFF media being the reference sample and *T. gondii* TUB1A being the housekeeping gene.

## SUPPLEMENTAL MATERIAL

Supplemental figure legends spectrum.03472-25-s0001.docx Figure legends for Fig. S1 to S11.

Fig. S1 spectrum.03472-25-s0002.tif Toxicity to host cells was not observed.

Fig. S2 spectrum.03472-25-s0003.tif Parasites grown in Complete Organoid media, A83-01 and SB202190, display a growth defect.

Fig. S3 spectrum.03472-25-s0004.tif DBA staining in infected cells treated with kinase inhibitors.

Fig. S4 spectrum.03472-25-s0005.tif DBA and abnormal nuclei quantitation after treating tachyzoite parasites with different medias.

Fig. S5 spectrum.03472-25-s0006.tif Morphological abnormalities following treatments at 24 hpi.

Fig. S6 spectrum.03472-25-s0007.tif qPCR assessment of stage-specific markers -: additional experimental replicates.

Fig. S7 spectrum.03472-25-s0008.tif GRA11B protein expression was not observed after infection with tachyzoites.

Supplemental figures spectrum.03472-25-s0009.docx Fig. S8 to S11.

ASM does not own the copyrights to Supplemental Material that may be linked to, or accessed through, an article. The authors have granted ASM a non-exclusive, world-wide license to publish the Supplemental Material files. Please contact the corresponding author directly for reuse.

## References

[1] Dubey JP, Frenkel JK. 1972. Cyst-induced toxoplasmosis in cats. J Protozool 19:155–177. doi:10.1111/j.1550-7408.1972.tb03431.x 5008846

[2] Ramakrishnan C, Maier S, Walker RA, Rehrauer H, Joekel DE, Winiger RR, Basso WU, Grigg ME, Hehl AB, Deplazes P, Smith NC. 2019. An experimental genetically attenuated live vaccine to prevent transmission of Toxoplasma gondii by cats. Sci Rep 9:1474. doi:10.1038/s41598-018-37671-8 30728393 PMC6365665

[3] Martorelli Di Genova B, Wilson SK, Dubey JP, Knoll LJ. 2019. Intestinal delta-6-desaturase activity determines host range for Toxoplasma sexual reproduction. PLoS Biol 17:e3000364. doi:10.1371/journal.pbio.3000364 31430281 PMC6701743

[4] Miura S, Suzuki A. 2018. Brief summary of the current protocols for generating intestinal organoids. Dev Growth Differ 60:387–392. doi:10.1111/dgd.12559 30039581

[5] Chen L, Qiu X, Dupre A, Pellon-Cardenas O, Fan X, Xu X, Rout P, Walton KD, Burclaff J, Zhang R, Fang W, Ofer R, Logerfo A, Vemuri K, Bandyopadhyay S, Wang J, Barbet G, Wang Y, Gao N, Perekatt AO, Hu W, Magness ST, Spence JR, Verzi MP. 2023. TGFB1 induces fetal reprogramming and enhances intestinal regeneration. Cell Stem Cell 30:1520–1537. doi:10.1016/j.stem.2023.09.015 37865088 PMC10841757

[6] Tsai Y-H, Wu A, Wu JH, Capeling MM, Holloway EM, Huang S, Czerwinkski M, Glass I, Higgins PDR, Spence JR. 2022. Acquisition of NOTCH dependence is a hallmark of human intestinal stem cell maturation. Stem Cell Reports 17:1138–1153. doi:10.1016/j.stemcr.2022.03.007 35395175 PMC9133587

[7] Oost MJ, Ijaz A, van Haarlem DA, van Summeren K, Velkers FC, Kraneveld AD, Venema K, Jansen CA, Pieters RHH, Ten Klooster JP. 2022. Chicken-derived RSPO1 and WNT3 contribute to maintaining longevity of chicken intestinal organoid cultures. Sci Rep 12:10563. doi:10.1038/s41598-022-14875-7 35732901 PMC9217957

[8] Mudaliar P, Pradeep P, Abraham R, Sreekumar E. 2021. Targeting cap-dependent translation to inhibit Chikungunya virus replication: selectivity of p38 MAPK inhibitors to virus-infected cells due to autophagy-mediated down regulation of phospho-ERK. J Gen Virol 102:001629. doi:10.1099/jgv.0.001629 34328830

[9] Brunetti JE, Quintana VM, Scolaro LA, Castilla V. 2022. Inhibitors of the p38 cell signaling pathway as antiviral compounds against Junín virus. Arch Virol 167:935–940. doi:10.1007/s00705-022-05388-9 35133480 PMC8852809

[10] Johnson RA, Huong S-M, Huang E-S. 1999. Inhibitory effect of 4-(4-fluorophenyl)-2-(4-hydroxyphenyl)-5-(4-pyridyl)1H - imidazole on HCMV DNA replication and permissive infection. Antiviral Res 41:101–111. doi:10.1016/s0166-3542(99)00002-9 10320043

[11] Wei S, Marches F, Daniel B, Sonda S, Heidenreich K, Curiel T. 2002. Pyridinylimidazole p38 mitogen-activated protein kinase inhibitors block intracellular Toxoplasma gondii replication. Int J Parasitol 32:969–977. doi:10.1016/s0020-7519(02)00061-9 12076626

[12] Wei S, Daniel BJ, Brumlik MJ, Burow ME, Zou W, Khan IA, Wadsworth S, Siekierka J, Curiel TJ. 2007. Drugs designed to inhibit human p38 mitogen-activated protein kinase activation treat Toxoplasma gondii and Encephalitozoon cuniculi infection. Antimicrob Agents Chemother 51:4324–4328. doi:10.1128/AAC.00680-07 17923491 PMC2168015

[13] Brown KM, Suvorova E, Farrell A, McLain A, Dittmar A, Wiley GB, Marth G, Gaffney PM, Gubbels MJ, White M, Blader IJ. 2014. Forward genetic screening identifies a small molecule that blocks Toxoplasma gondii growth by inhibiting both host- and parasite-encoded kinases. PLoS Pathog 10:e1004180. doi:10.1371/journal.ppat.1004180 24945800 PMC4055737

[14] Ettayebi K, Crawford SE, Murakami K, Broughman JR, Karandikar U, Tenge VR, Neill FH, Blutt SE, Zeng X-L, Qu L, Kou B, Opekun AR, Burrin D, Graham DY, Ramani S, Atmar RL, Estes MK. 2016. Replication of human noroviruses in stem cell–derived human enteroids. Science 353:1387–1393. doi:10.1126/science.aaf5211 27562956 PMC5305121

[15] Koshy AA, Dietrich HK, Christian DA, Melehani JH, Shastri AJ, Hunter CA, Boothroyd JC. 2012. Toxoplasma co-opts host cells it does not invade. PLoS Pathog 8:e1002825. doi:10.1371/journal.ppat.1002825 22910631 PMC3406079

[16] Ramakrishnan C, Walker RA, Eichenberger RM, Hehl AB, Smith NC. 2017. The merozoite-specific protein, TgGRA11B, identified as a component of the Toxoplasma gondii parasitophorous vacuole in a tachyzoite expression model. Int J Parasitol 47:597–600. doi:10.1016/j.ijpara.2017.04.001 28526607

[17] Chou T-C, Talalay P. 1984. Quantitative analysis of dose-effect relationships: the combined effects of multiple drugs or enzyme inhibitors. Adv Enzyme Regul 22:27–55. doi:10.1016/0065-2571(84)90007-4 6382953

[18] Chou T-C. 2006. Theoretical basis, experimental design, and computerized simulation of synergism and antagonism in drug combination studies. Pharmacol Rev 58:621–681. doi:10.1124/pr.58.3.10 16968952

[19] Ramírez-Flores CJ, Hryckowian ND, Gale AN, Babatunde KA, Lares M, Beebe DJ, Kerr SC, Knoll LJ. 2025. Modeling Toxoplasma gondii-gut early interactions using a human microphysiological system. PLoS Negl Trop Dis 19:e0012855. doi:10.1371/journal.pntd.0012855 39903779 PMC12136440

[20] Paredes-Santos TC, Tomita T, Yan Fen M, de Souza W, Attias M, Vommaro RC, Weiss LM. 2016. Development of dual fluorescent stage specific reporter strain of Toxoplasma gondii to follow tachyzoite and bradyzoite development in vitro and in vivo. Microbes Infect 18:39–47. doi:10.1016/j.micinf.2015.09.016 26432517 PMC4715970

[21] Davies SP, Reddy H, Caivano M, Cohen P. 2000. Specificity and mechanism of action of some commonly used protein kinase inhibitors. Biochem J 351:95–105. doi:10.1042/0264-6021:3510095 10998351 PMC1221339

[22] Tojo M, Hamashima Y, Hanyu A, Kajimoto T, Saitoh M, Miyazono K, Node M, Imamura T. 2005. The ALK-5 inhibitor A-83-01 inhibits Smad signaling and epithelial-to-mesenchymal transition by transforming growth factor-beta. Cancer Sci 96:791–800. doi:10.1111/j.1349-7006.2005.00103.x 16271073 PMC11159601

[23] Sidik SM, Huet D, Ganesan SM, Huynh M-H, Wang T, Nasamu AS, Thiru P, Saeij JPJ, Carruthers VB, Niles JC, Lourido S. 2016. A genome-wide CRISPR screen in toxoplasma identifies essential apicomplexan genes. Cell 166:1423–1435. doi:10.1016/j.cell.2016.08.019 27594426 PMC5017925

[24] Suvorova ES, Francia M, Striepen B, White MW. 2015. A novel bipartite centrosome coordinates the apicomplexan cell cycle. PLoS Biol 13:e1002093. doi:10.1371/journal.pbio.1002093 25734885 PMC4348508

[25] Brown KM, Long S, Sibley LD. 2018. Conditional knockdown of proteins using auxin-inducible degron (AID) fusions in Toxoplasma gondii. Bio Protoc 8:e2728. doi:10.21769/BioProtoc.2728 PMC5890294 29644255

[26] Ulu A, Srivastava S, Kachour N, White MW, Wilson EH. 2025. Bradyzoite subtypes rule the crossroads of Toxoplasma development. bioRxiv:2025.08.23.671938. doi:10.1101/2025.08.23.671938 PMC12917143 41580398

[27] Vizcarra EA, Goerner AL, Ulu A, Hong DD, Bergersen KV, Talavera MA, Le Roch K, Wilson EH, White MW. 2023. An ex vivo model of Toxoplasma recrudescence reveals developmental plasticity of the bradyzoite stage. mBio 14:e01836–23. doi:10.1128/mbio.01836-23 37675999 PMC10653814

[28] Donald RGK, Allocco J, Singh SB, Nare B, Salowe SP, Wiltsie J, Liberator PA. 2002. Toxoplasma gondii cyclic GMP-dependent kinase: chemotherapeutic targeting of an essential parasite protein kinase. Eukaryot Cell 1:317–328. doi:10.1128/EC.1.3.317-328.2002 12455981 PMC118020

[29] Radke JR, Donald RG, Eibs A, Jerome ME, Behnke MS, Liberator P, White MW. 2006. Changes in the expression of human cell division autoantigen-1 influence Toxoplasma gondii growth and development. PLoS Pathog 2:e105. doi:10.1371/journal.ppat.0020105 17069459 PMC1626100

[30] Behnke MS, Radke JB, Smith AT, Sullivan WJ Jr, White MW. 2008. The transcription of bradyzoite genes in Toxoplasma gondii is controlled by autonomous promoter elements. Mol Microbiol 68:1502–1518. doi:10.1111/j.1365-2958.2008.06249.x 18433450 PMC2440561

[31] Saksouk N, Bhatti MM, Kieffer S, Smith AT, Musset K, Garin J, Sullivan WJ, Cesbron-Delauw M-F, Hakimi M-A. 2005. Histone-modifying complexes regulate gene expression pertinent to the differentiation of the protozoan parasite Toxoplasma gondii. Mol Cell Biol 25:10301–10314. doi:10.1128/MCB.25.23.10301-10314.2005 16287846 PMC1291236

[32] Bougdour A, Maubon D, Baldacci P, Ortet P, Bastien O, Bouillon A, Barale J-C, Pelloux H, Ménard R, Hakimi M-A. 2009. Drug inhibition of HDAC3 and epigenetic control of differentiation in Apicomplexa parasites. J Exp Med 206:953–966. doi:10.1084/jem.20082826 19349466 PMC2715132

[33] Dubey JP. 1996. Infectivity and pathogenicity of Toxoplasma gondii oocysts for cats. J Parasitol 82:957–961. 8973406

[34] Dubey JP, Frenkel JK. 1976. Feline toxoplasmosis from acutely infected mice and the development of Toxoplasma cysts. J Protozool 23:537–546. doi:10.1111/j.1550-7408.1976.tb03836.x 1003342

[35] Freyre A, Dubey JP, Smith DD, Frenkel JK. 1989. Oocyst-induced Toxoplasma gondii infections in cats. J Parasitol 75:750–755. 2795377

[36] Lindner SE, Swearingen KE, Shears MJ, Walker MP, Vrana EN, Hart KJ, Minns AM, Sinnis P, Moritz RL, Kappe SHI. 2019. Transcriptomics and proteomics reveal two waves of translational repression during the maturation of malaria parasite sporozoites. Nat Commun 10:4964. doi:10.1038/s41467-019-12936-6 31673027 PMC6823429

[37] Holmes M, Itaas V, Ananvoranich S. 2014. Sustained translational repression of lactate dehydrogenase 1 in Toxoplasma gondii bradyzoites is conferred by a small regulatory RNA hairpin. FEBS J 281:5077–5091. doi:10.1111/febs.13048 25223457

[38] Rooney PJ, Neal LM, Knoll LJ. 2011. Involvement of a Toxoplasma gondii chromatin remodeling complex ortholog in developmental regulation. PLoS One 6:e19570. doi:10.1371/journal.pone.0019570 21655329 PMC3104990

[39] Lunghi M, Galizi R, Magini A, Carruthers VB, Di Cristina M. 2015. Expression of the glycolytic enzymes enolase and lactate dehydrogenase during the early phase of Toxoplasma differentiation is regulated by an intron retention mechanism. Mol Microbiol 96:1159–1175. doi:10.1111/mmi.12999 25777509

