# Supplementary material for: Kinase inhibitors in organoid media influence Toxoplasma gondii growth and development
Source: Microbiol Spectr. 2026 Apr 3;14(5):e03472-25. doi: 10.1128/spectrum.03472-25 (PMC13141834; doi:10.1128/spectrum.03472-25)
Supplement: Supplemental figure legends — Figure legends for Fig. S1 to S11. [file spectrum.03472-25-s0001.docx]

**Supplemental Figure Legends**

**Fig. S1.** Toxicity to host cells was not observed. HFFs were allowed to reach confluency then treated with the following: Advanced (ADV) DMEM, complete organoid media (COM ORG), advanced DMEM with 500 nM A83-01, or advanced DMEM with 10 µM SB202190. Shown is a representative set of images.

**Fig. S2.** Parasites grown in complete organoid media, A83-01 and SB202190 display a growth defect. A) HFFs were infected with the *T. gondii* strain PRU Cre mCherry at an MOI of 0.1. After 3 hpi, the media was removed and replaced with the conditions as shown in the legend and the plate was moved to an IncuCyte Live Cell Imaging system where red fluorescence and brightfield images were captured every 12 hours for 5 days. With infection as described in panel A, with varying concentrations of A83-01 (B) and SB202190 (C) in advanced DMEM.

**Fig. S3.** Cyst wall like structures are visible under some treatment conditions. HFFs were infected with the *T. gondii* strain ME49∆hpt luciferase at a MOI of 0.1. At 3 hpi, the media was removed and replaced with the following: complete organoid (COM ORG), advanced (ADV) DMEM, reduced organoid (RED ORG), or advanced DMEM plus two different concentrations of A83-01 and SB202190. At 4 dpi, cells were fixed with 100% cold methanol and stained with Pan *Toxoplasma* antibody (purple), *Dolichos biflorus* agglutinin (DBA, yellow), mounted in DAPI (blue), and imaged with differential interference contrast (DIC) microscopy. Shown is a representative set of images taken at the same magnification and the white size bar is 50 µm.

**Fig. S4.** Observational phenotypes are quantifiable, with the greatest effect seen at the highest doses. HFFs were infected with the *T. gondii* strain ME49∆hpt luciferase at a MOI of 0.1. At 3 hpi, the media was removed and replaced with the following: complete organoid (COM ORG), advanced (ADV) DMEM, reduced organoid (RED ORG), or advanced DMEM plus two different concentrations of A83-01 and SB202190. At 4 dpi, cells were fixed with 100% cold methanol and stained with Pan *Toxoplasma* antibody, *Dolichos biflorus* agglutinin (DBA), mounted in DAPI. A count of 50 vacuoles per condition was performed and each vacuole was scored accordingly: normal, +DBA, +abnormal nuclei, or both (+DBA and + abnormal nuclei). Data presented is the mean of 3 replicates as a % of the total (50). Error bars indicate standard error of the mean.

**Fig. S5.** A83-01 and SB202190 are still effective when added after the parasites have begun replicating. HFFs were infected with *T. gondii* strain ME49∆hpt luciferase at a MOI of 0.1. At 3 or 24 hpi, the media was removed and replaced with advanced DMEM plus A83-01 or SB202190. At 4 dpi, cells were fixed with 100% cold methanol and stained with Pan Toxoplasma antibody (purple), *Dolichos biflorus* agglutinin (DBA, yellow), mounted in DAPI (blue), and imaged with differential interference contrast (DIC) microscopy. Shown is a representative set of images taken at the same magnification and the white size bar is 50 µm.

**Fig. S6.** A83-01 and SB20190 promote bradyzoite and pre-sexual gene expression. HFFs were infected with the *T. gondii* strain ME49 ∆hpt luciferase at a MOI of 0.1. At 3 hpi, the media was removed and replaced with the following: DMEM with 10% FBS, advanced (ADV) DMEM, complete organoid (Org), reduced organoid (Org), or advanced DMEM plus two different concentrations of A83-01 and SB202190. At 4 dpi, monolayers were scraped, pelleted and frozen at -80C. Cell pellets were resuspended in TRIZOL, RNA was isolated, cDNA was produced, and qPCR performed using primers listed on Table 2. The internal sample control is *T. gondii* TUB1A and the external sample control is parasites grown in DMEM with 10% FBS. A one-way ANOVA was performed on the technical replicates with* ‎P ‎< 0.05, ** P < 0.01, *** P < 0.001, **** P < 0.0001.

**Fig. S7.** GRA11B protein expression was not observed under tachyzoite infection conditions by IFA. HFFs were infected with the *T. gondii* strain ME49∆hpt luciferase at a MOI of 0.1. At 3 hpi, the media was removed and replaced with the following: complete organoid (COM ORG), advanced (ADV) DMEM, reduced organoid (RED ORG), or advanced DMEM with 500 nM A83-01. At 4 dpi, cells were fixed with 100% cold methanol and stained with Pan *Toxoplasma* antibody (purple), GRA11B (yellow), mounted in DAPI (blue), and imaged with differential interference contrast (DIC) microscopy. Shown is a representative set of images taken at the same magnification and the white size bar is 50 µm.

**Fig. S8.** Observational phenotypes are quantifiable with the greatest effect seen at the high doses. Confluent HFFs were infected with in vitro bradyzoites from the *T. gondii* strain ME49 ∆hpt luciferase at an MOI of 0.5. At 3 hpi, the media was removed and replaced with the following: complete organoid (COM ORG), advanced (ADV) DMEM, reduced organoid (RED ORG), or advanced DMEM plus two different concentrations of A83-01 and SB202190. At 7 dpi cells were fixed with 100% cold methanol and stained with Pan *Toxoplasma* antibody, *Dolichos biflorus* agglutinin (DBA), mounted in DAPI. A) Raw counts of 3 coverslips (ADV DMEM and SB202190 Low), 4 coverslips (Reduced Org, A83-01 Low), 7 coverslips (A83-01 High), and 8 coverslips (Complete Org, SB202190 H). B) % of the total vacuoles counted in A.

**Fig. S9.** Growth curves reflect a maintenance of bradyzoite characteristics under drug treatment. HFFs were infected with in vitro bradyzoites from the *T. gondii* strain EGS. After 3 hpi, the media was removed and replaced with the following: advanced (ADV) DMEM, complete organoid (Org), reduced organoid (Org), or advanced DMEM plus two different concentrations of A83-01 and SB202190. The plate was subsequently moved to an IncuCyte Live Cell Imaging system where red fluorescence, green fluorescence and brightfield images were captured every 12 hours for 6 days. Panel A is a paired replicate of GFP and mCherry fluorescence and panel B is another paired replicate of GFP and mCherry fluorescence. Statistical analysis was performed using a one-way ANOVA followed by post hoc Dunnett’s test comparing the area under the curve of ADV DMEM media to all other media conditions. * Indicates P < 0.05, ** indicates P < 0.01, *** indicates P < 0.001, **** indicates P < 0.0001

**Fig. S10.** High doses of A83-01 and SB202190 promote the maintenance of the bradyzoites stage and prime for transition to pre-sexual stages. HFFs were grown to confluency and infected with in vitro bradyzoites of the *T. gondii* strain ME49 ∆hpt luciferase at a MOI of 0.5. After 3 hpi the media was removed and replaced with the following: advanced (ADV) DMEM, complete organoid (Org), reduced organoid (Org), or advanced DMEM plus two different concentrations of A83-01(A83) and SB202190 (SB190). At 5-6 dpi, monolayers were scraped, pelleted and frozen at -80C. Cell pellets were resuspended in TRIZOL, RNA was isolated, cDNA was produced, and qPCR performed using primers listed on Table 2. The internal sample control is *T. gondii* TUB1A and the external sample control is parasites grown in DMEM with 10% FBS. A one-way ANOVA was performed on the technical replicates with * ‎P ‎< 0.05, ** P < 0.01, *** P < 0.001, **** P < 0.0001.

**Fig. S11.** GRA11B protein expression was not observed under bradyzoite infection conditions by IFA. Confluent HFFs were infected with in vitro bradyzoites from the *T. gondii* strain ME49 ∆hpt luciferase at an MOI of 0.5. At 3 hpi, the media was removed and replaced with the following: complete organoid (COM ORG), advanced (ADV) DMEM, reduced organoid (RED ORG), or advanced DMEM with 500 nM A83-01. At 4 dpi, cells were fixed with 100% cold methanol and stained with Pan *Toxoplasma* antibody (purple), GRA11B (yellow), mounted in DAPI (blue), and imaged with differential interference contrast (DIC) microscopy. Shown is a representative set of images taken at the same magnification and the white size bar is 50 µm.
