# Supplementary figures and images for: Kinase inhibitors in organoid media influence Toxoplasma gondii growth and development
Source: Microbiol Spectr. 2026 Apr 3;14(5):e03472-25. doi: 10.1128/spectrum.03472-25 (PMC13141834; doi:10.1128/spectrum.03472-25)

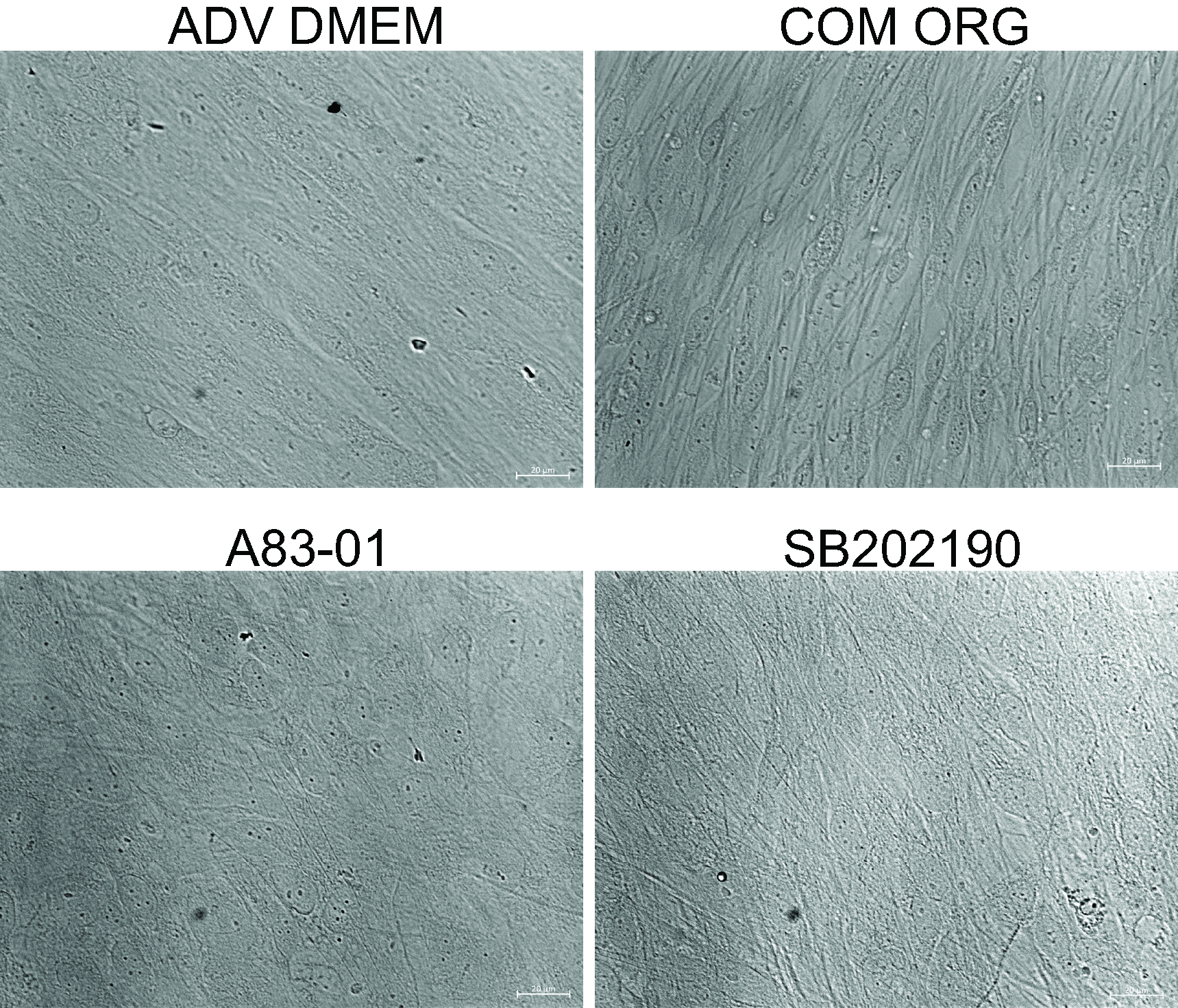

Supplement: Fig. S1 — Toxicity to host cells was not observed. [file spectrum.03472-25-s0002.tif]

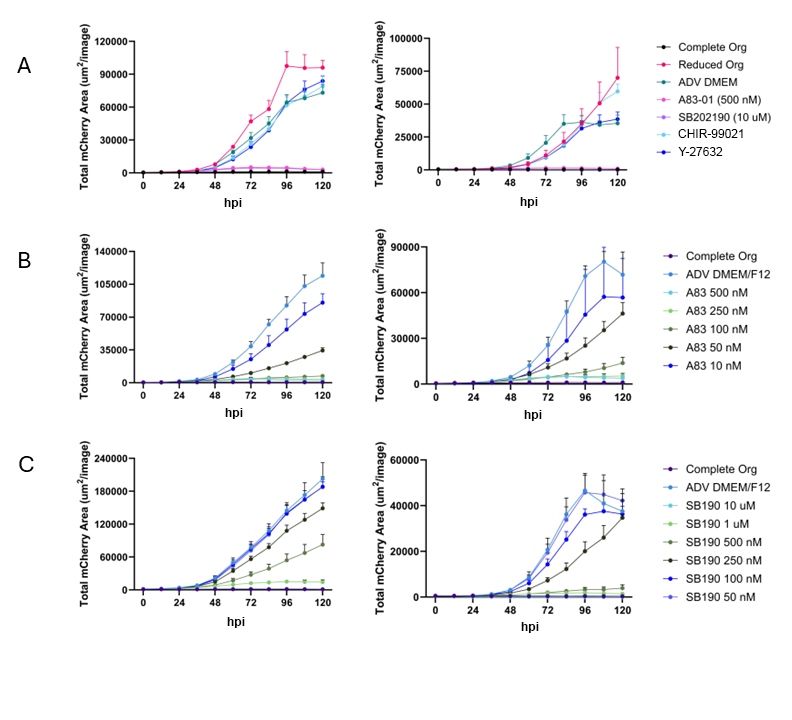

Supplement: Fig. S2 — Parasites grown in Complete Organoid media, A83-01 and SB202190, display a growth defect. [file spectrum.03472-25-s0003.tif]

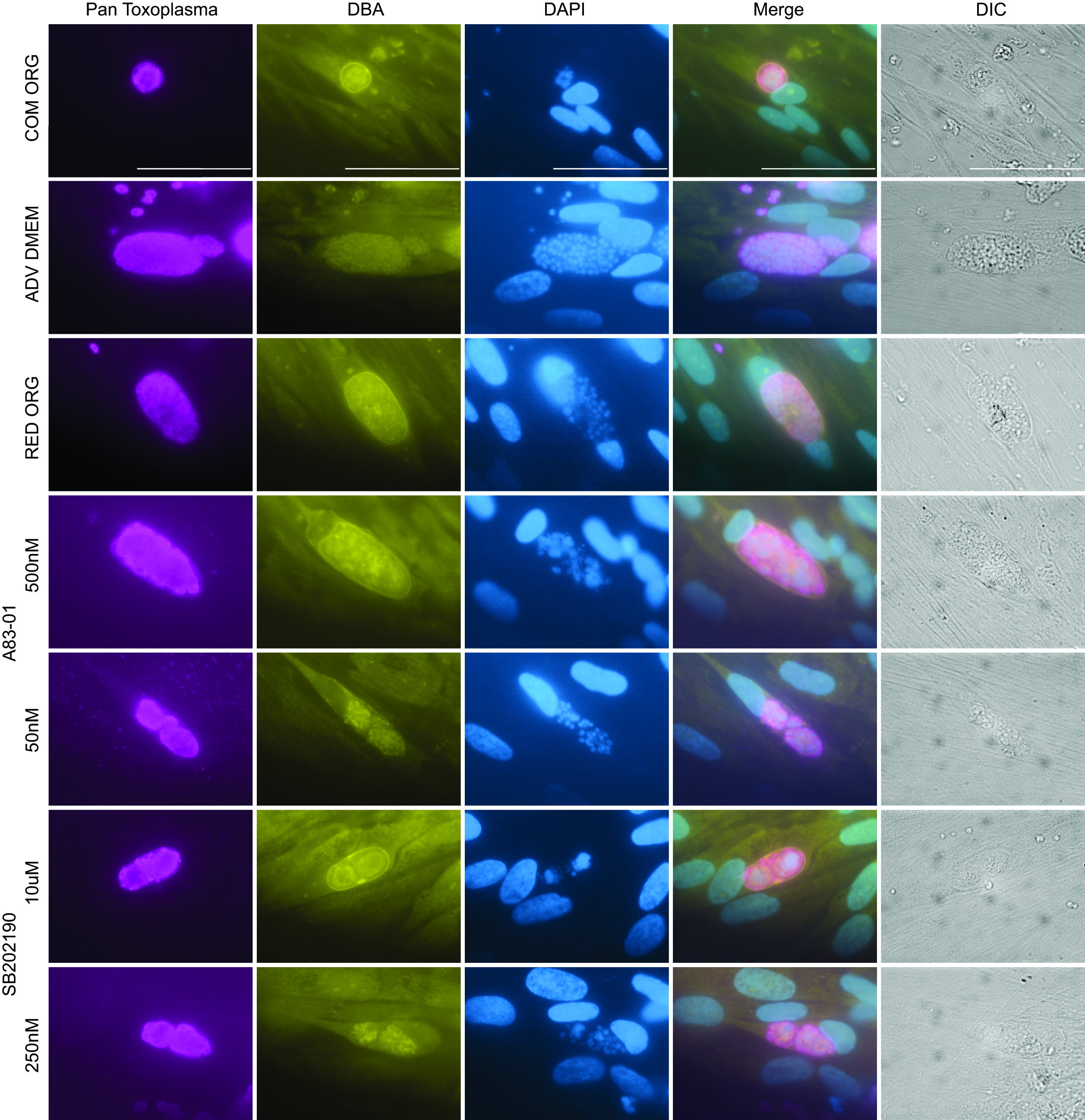

Supplement: Fig. S3 — DBA staining in infected cells treated with kinase inhibitors. [file spectrum.03472-25-s0004.tif]

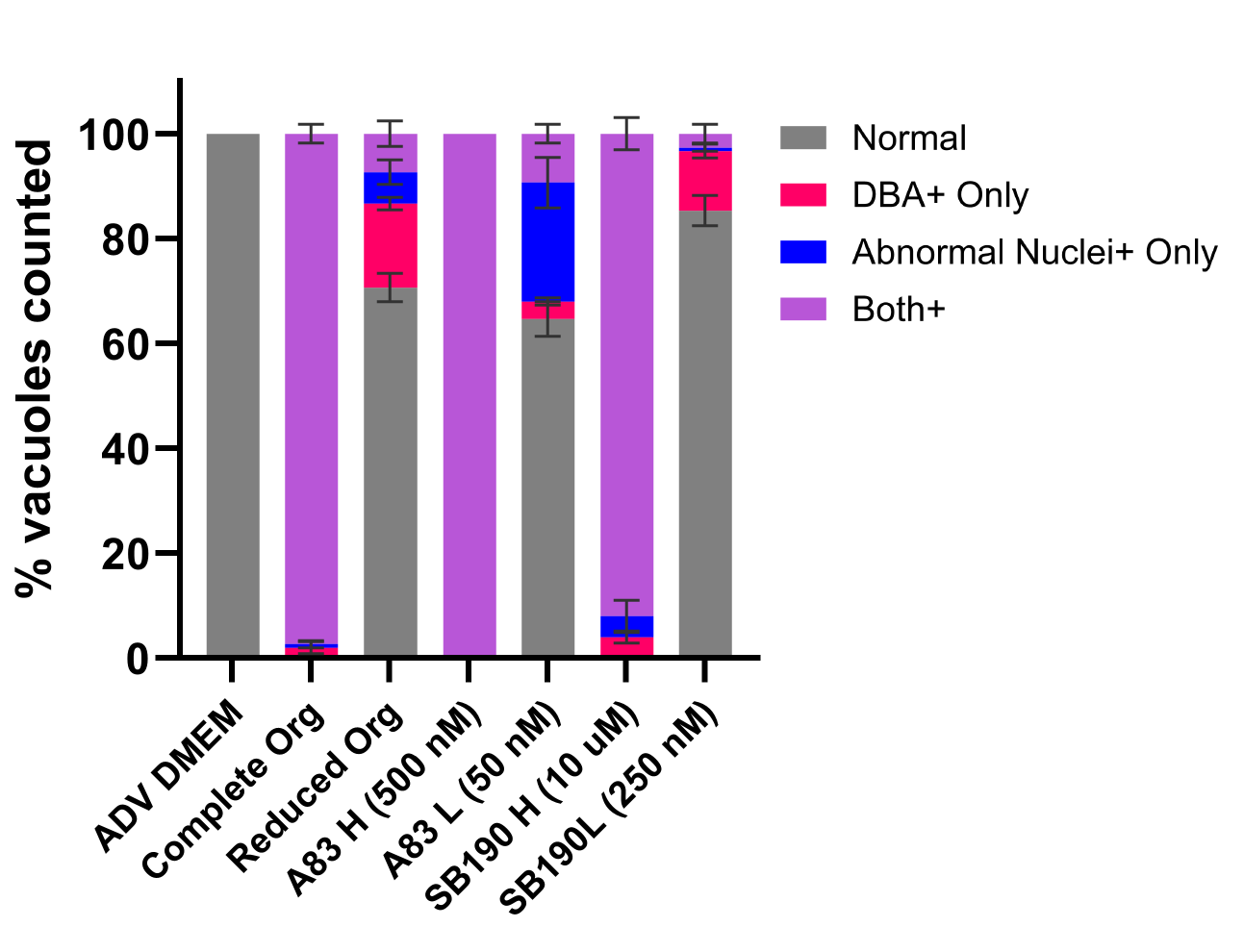

Supplement: Fig. S4 — DBA and abnormal nuclei quantitation after treating tachyzoite parasites with different medias. [file spectrum.03472-25-s0005.tif]

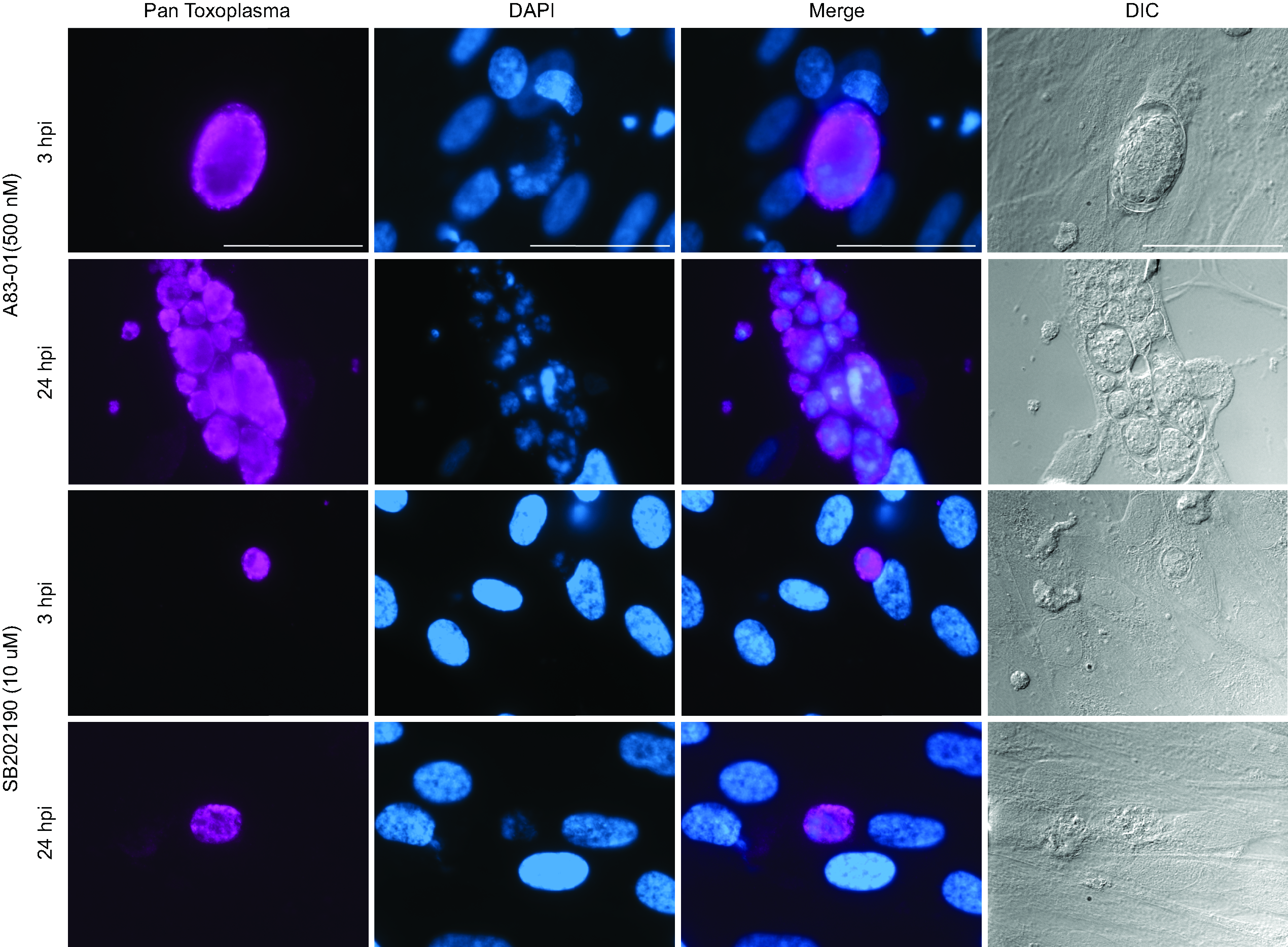

Supplement: Fig. S5 — Morphological abnormalities following treatments at 24 hpi. [file spectrum.03472-25-s0006.tif]

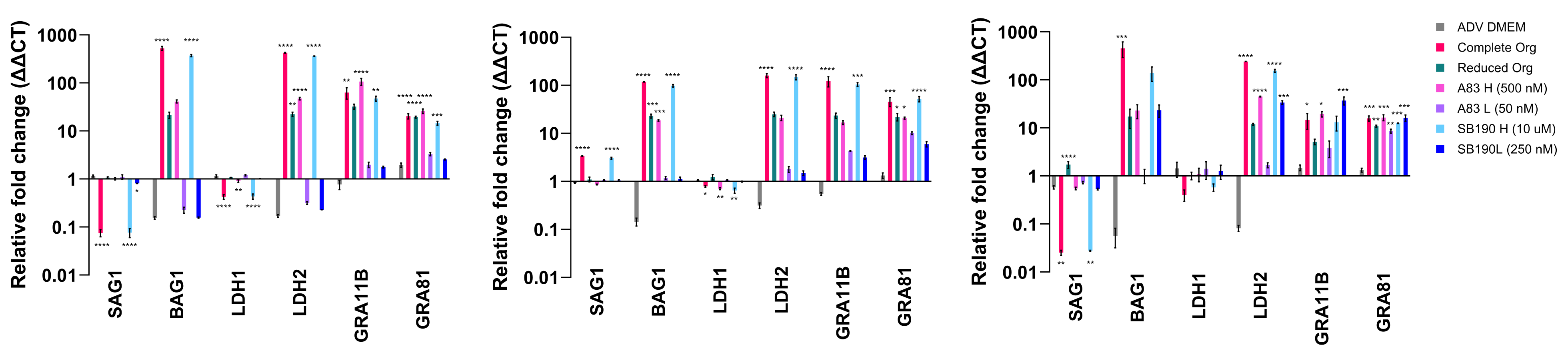

Supplement: Fig. S6 — qPCR assessment of stage-specific markers -: additional experimental replicates. [file spectrum.03472-25-s0007.tif]

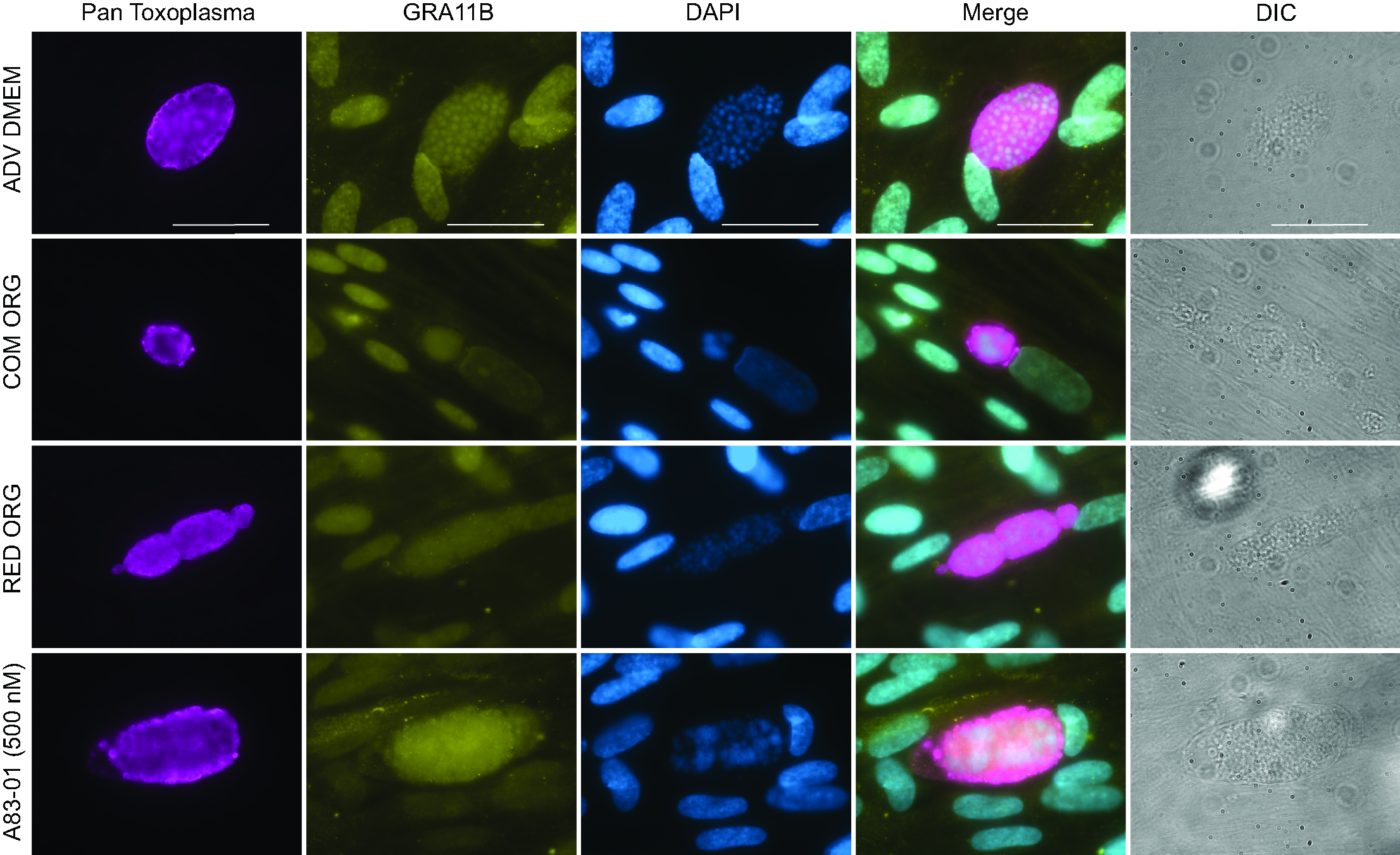

Supplement: Fig. S7 — GRA11B protein expression was not observed after infection with tachyzoites. [file spectrum.03472-25-s0008.tif]
