# Supplementary material for: Kinase inhibitors in organoid media influence Toxoplasma gondii growth and development
Source: Microbiol Spectr. 2026 Apr 3;14(5):e03472-25. doi: 10.1128/spectrum.03472-25 (PMC13141834; doi:10.1128/spectrum.03472-25)
Supplement: Supplemental figures — Fig. S8 to S11. [file spectrum.03472-25-s0009.docx]

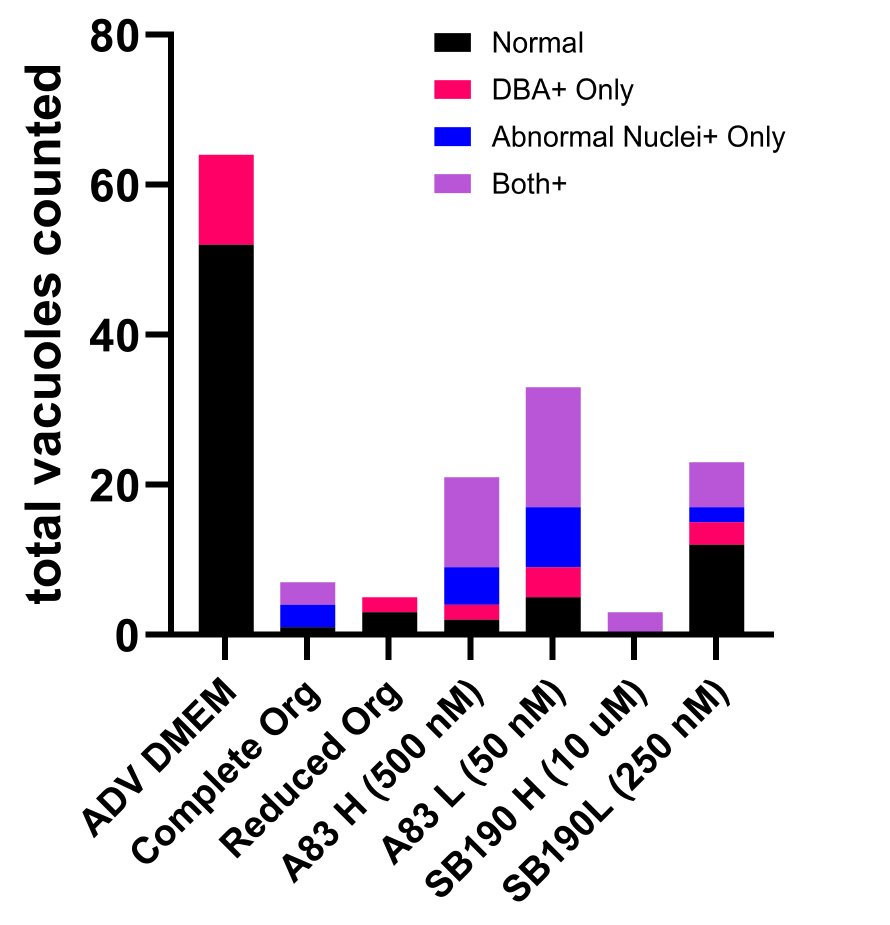


**Fig. S8.** Observational phenotypes are quantifiable with the greatest effect seen at the high doses. Confluent HFFs were infected with in vitro bradyzoites from the *T. gondii* strain ME49 ∆hpt luciferase at an MOI of 0.5. At 3 hpi, the media was removed and replaced with the following: complete organoid (COM ORG), advanced (ADV) DMEM, reduced organoid (RED ORG), or advanced DMEM plus two different concentrations of A83-01 and SB202190. At 7 dpi cells were fixed with 100% cold methanol and stained with Pan *Toxoplasma* antibody, *Dolichos biflorus* agglutinin (DBA), mounted in DAPI. A) Raw counts of 3 coverslips (ADV DMEM and SB202190 Low), 4 coverslips (Reduced Org, A83-01 Low), 7 coverslips (A83-01 High), and 8 coverslips (Complete Org, SB202190 H).


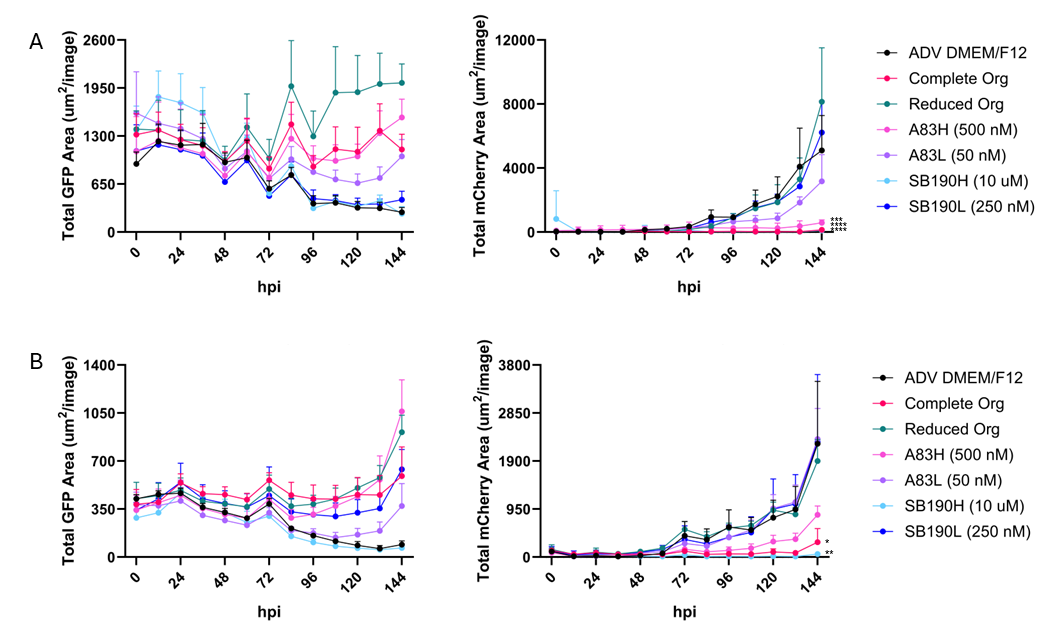


**Fig. S9.** Growth curves reflect a maintenance of bradyzoite characteristics under drug treatment. HFFs were infected with in vitro bradyzoites from the *T. gondii* strain EGS. After 3 hpi, the media was removed and replaced with the following: advanced (ADV) DMEM, complete organoid (Org), reduced organoid (Org), or advanced DMEM plus two different concentrations of A83-01 and SB202190. The plate was subsequently moved to an IncuCyte Live Cell Imaging system where red fluorescence, green fluorescence and brightfield images were captured every 12 hours for 6 days. Panel A is a paired replicate of GFP and mCherry fluorescence and panel B is another paired replicate of GFP and mCherry fluorescence. Statistical analysis was performed using a one-way ANOVA followed by post hoc Dunnett’s test comparing the area under the curve of ADV DMEM media to all other media conditions. * Indicates P < 0.05, ** indicates P < 0.01, *** indicates P < 0.001, **** indicates P < 0.0001.


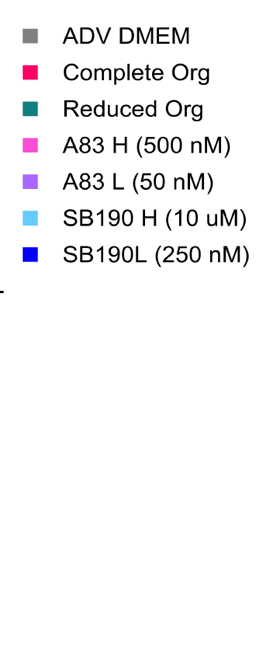

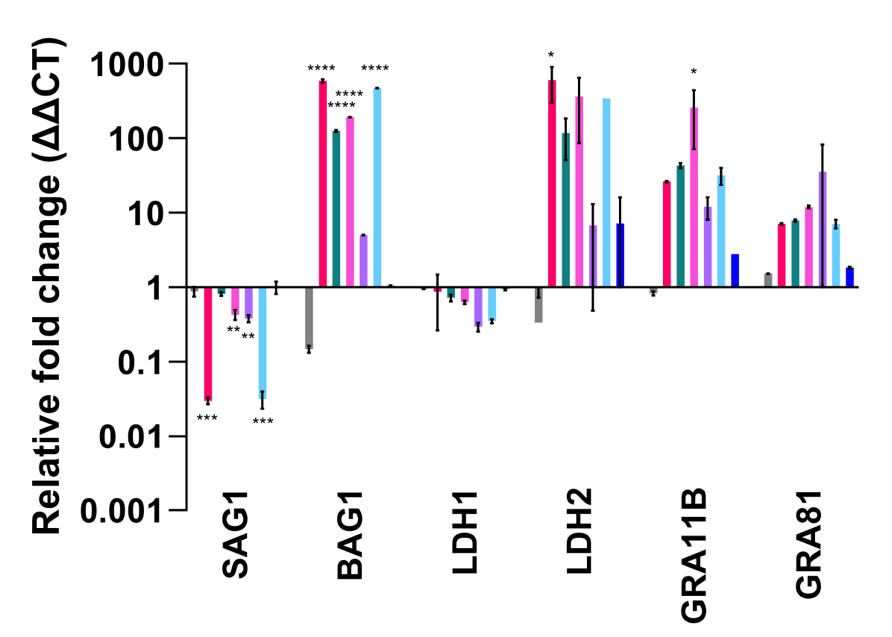


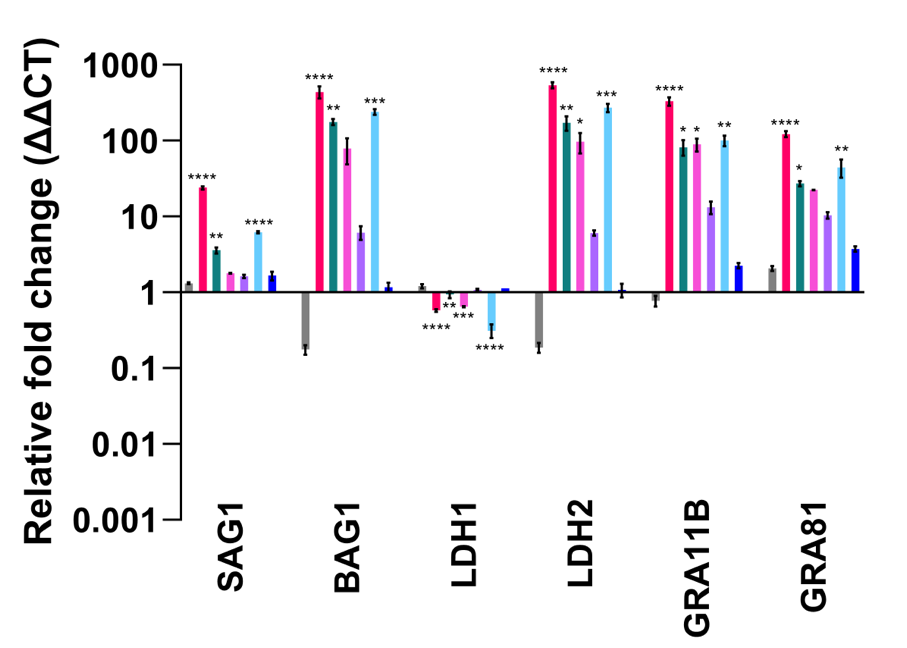


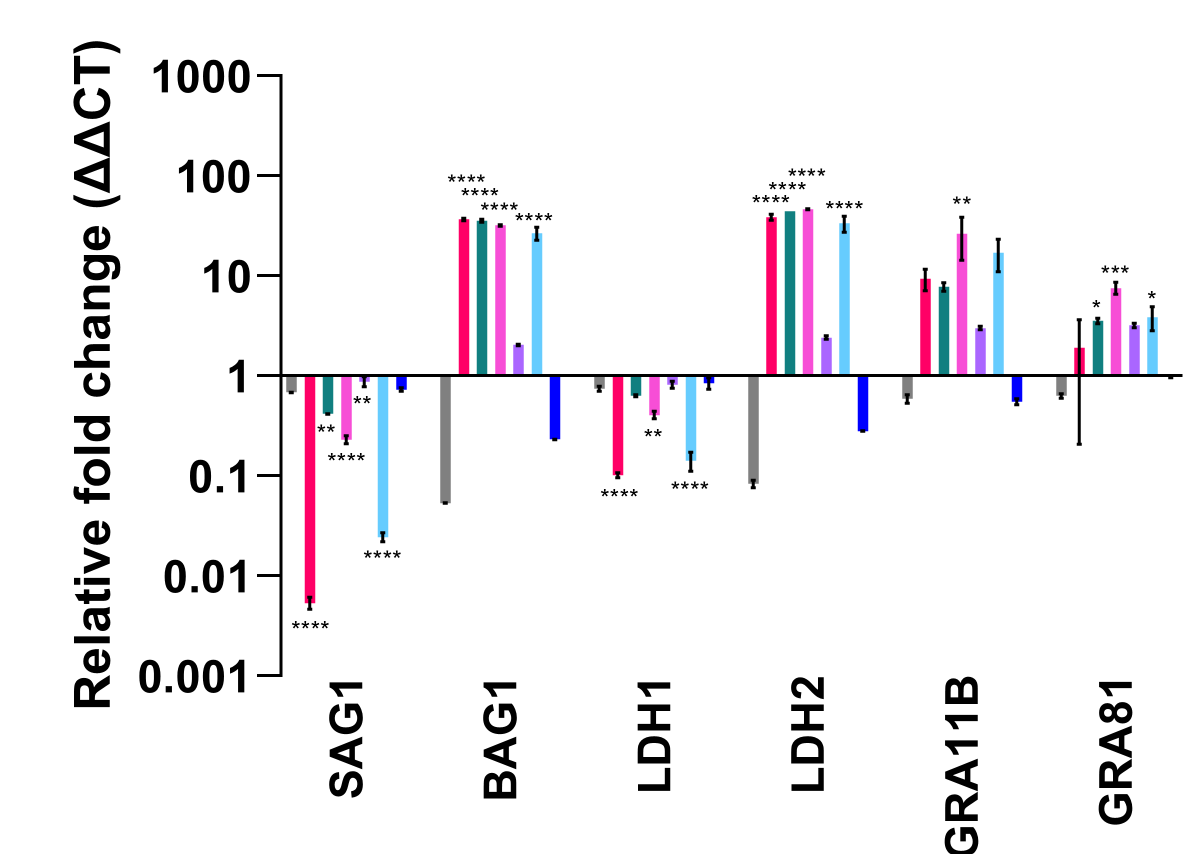


**Fig. S10.** High doses of A83-01 and SB202190 promote the maintenance of the bradyzoites stage and prime for transition to pre-sexual stages. HFFs were grown to confluency and infected with in vitro bradyzoites of the *T. gondii* strain ME49 ∆hpt luciferase at a MOI of 0.5. After 3 hpi the media was removed and replaced with the following: advanced (ADV) DMEM, complete organoid (Org), reduced organoid (Org), or advanced DMEM plus two different concentrations of A83-01(A83) and SB202190 (SB190). At 5-6 dpi, monolayers were scraped, pelleted and frozen at -80C. Cell pellets were resuspended in TRIZOL, RNA was isolated, cDNA was produced, and qPCR performed using primers listed on Table 2. The internal sample control is *T. gondii* TUB1A and the external sample control is parasites grown in DMEM with 10% FBS. A one-way ANOVA was performed on the technical replicates with * ‎P ‎< 0.05, ** P < 0.01, *** P < 0.001, **** P < 0.0001.


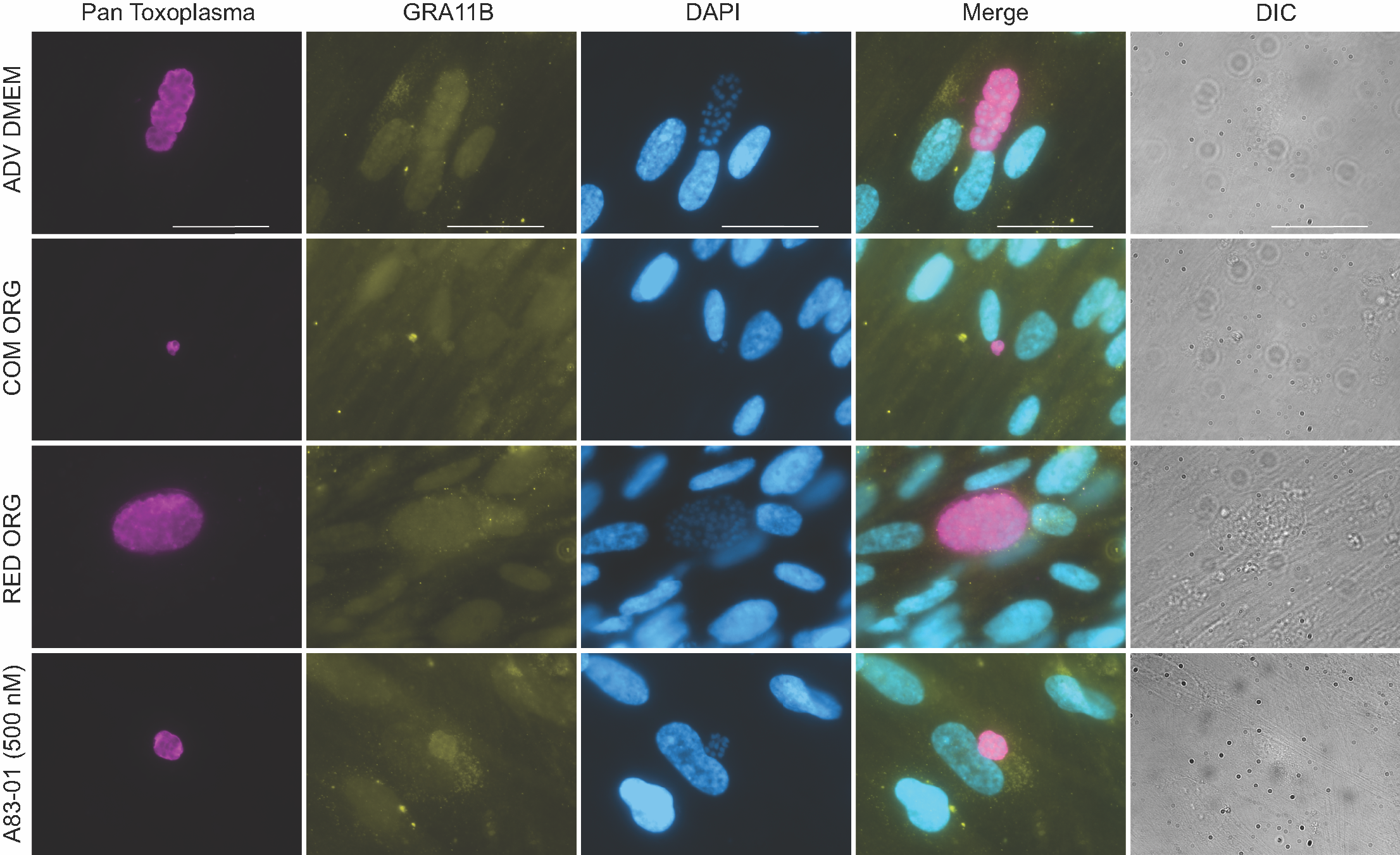


**Fig. S11.** GRA11B protein expression was not observed under bradyzoite infection conditions by IFA. Confluent HFFs were infected with in vitro bradyzoites from the *T. gondii* strain ME49 ∆hpt luciferase at an MOI of 0.5. At 3 hpi, the media was removed and replaced with the following: complete organoid (COM ORG), advanced (ADV) DMEM, reduced organoid (RED ORG), or advanced DMEM with 500 nM A83-01. At 4 dpi, cells were fixed with 100% cold methanol and stained with Pan *Toxoplasma* antibody (purple), GRA11B (yellow), mounted in DAPI (blue), and imaged with differential interference contrast (DIC) microscopy. Shown is a representative set of images taken at the same magnification and the white size bar is 50 µm.
